# RBM45 associates with nuclear stress bodies and forms nuclear inclusions during chronic cellular stress and in neurodegenerative diseases

**DOI:** 10.1186/s40478-020-00965-y

**Published:** 2020-06-26

**Authors:** Mahlon Collins, Yang Li, Robert Bowser

**Affiliations:** 1 Departments of Neurobiology and Neurology, Barrow Neurological Institute, Phoenix, AZ USA; 2 Department of Neurobiology, University of Pittsburgh, Pittsburgh, PA USA; 3 Current Address: Department of Genetics, Cell Biology, and Development, University of Minnesota, Minneapolis, MN USA; 4 CCurrent Address: School of Molecular Sciences, Arizona State University, Tempe, AZ USA

**Keywords:** RBM45, Nuclear stress bodies, Protein inclusions, Frontotemporal lobar degeneration, Amyotrophic lateral sclerosis, RNA binding proteins

## Abstract

The RNA binding protein (RBP) RBM45 forms nuclear and cytoplasmic inclusions in neurons and glia in amyotrophic lateral sclerosis (ALS), frontotemporal lobar degeneration with TDP-43 proteinopathy (FTLD-TDP), and Alzheimer’s disease (AD). The normal functions of RBM45 are poorly understood, as are the mechanisms by which it forms inclusions in disease. To better understand the normal and pathological functions of RBM45, we evaluated whether the protein functions via association with several membraneless organelles and whether such an association could promote the formation of nuclear RBM45 inclusions. Under basal conditions, RBM45 is diffusely distributed throughout the nucleus and does not localize to membraneless organelles, including nuclear speckles, Cajal bodies, or nuclear gems. During cellular stress, however, nuclear RBM45 undergoes a reversible, RNA-binding dependent incorporation into nuclear stress bodies (NSBs). Chronic stress leads to the persistent association of RBM45 with NSBs and the irreversible accumulation of nuclear RBM45 inclusions. We also quantified the cell type- and disease-specific patterns of RBM45 pathology in ALS, FTLD-TDP, and AD. RBM45 nuclear and cytoplasmic inclusions are found in both neurons and glia in ALS, FTLD-TDP, and AD but are absent in non-neurologic disease controls. Across neurodegenerative diseases, RBM45 nuclear inclusion pathology occurs more frequently than cytoplasmic RBM45 inclusion pathology and exhibits cell type-specific variation. Collectively, our results define new stress-associated functions of RBM45, a mechanism for nuclear RBM45 inclusion formation, a role for NSBs in the pathogenesis of ALS, FTLD-TDP, and AD, and further underscore the importance of protein self-association to both the normal and pathological functions of RBPs in these diseases.

## Introduction

RBM45 is a developmentally regulated RNA binding protein (RBP) that forms nuclear and cytoplasmic inclusions in neurons and glia in amyotrophic lateral sclerosis (ALS), frontotemporal lobar degeneration with TDP-43 proteinopathy (FTLD-TDP), and Alzheimer’s disease (AD) [1, 2]. Nuclear and cytoplasmic RBM45 inclusions have distinct compositions and morphologies. Cytoplasmic RBM45 inclusions contain TDP-43 and ubiquitin and exhibit a skein-like or globular morphology. In contrast, nuclear RBM45 inclusions are punctate in appearance and are negative for other disease-linked RBPs, including TDP-43 and FUS [1]. Aside from these unique properties, however, little is known about nuclear RBM45 inclusions. In particular, the mechanisms leading to their formation, their impact on cellular viability, and the cell-type specific patterns of nuclear RBM45 pathology across diseases remain unknown.

To better understand RBM45 inclusion formation, we and others have begun to characterize the normal functions of RBM45. The first efforts to this end focused on identifying functional domains in the RBM45 protein. By virtue of a bipartite nuclear localization sequence (NLS), RBM45 is a predominantly nuclear protein under basal conditions [3–5]. Mutation of the RBM45 NLS leads to cytoplasmic sequestration of the protein, with attendant incorporation into cytoplasmic stress granules (SGs) [4, 5]. RBM45 also contains 3 RNA recognition motifs (RRMs) that govern its binding to RNA sequences, with a GC-rich sequence preference [4–6]. Finally, RBM45 contains a novel “homo-oligomer assembly” (HOA) domain, an internal intrinsically disordered region that mediates the ability of RBM45 to self-associate and interact with other RBPs (including TDP-43 and FUS) [4, 6, 7]. Our studies of the HOA domain demonstrated that cellular RBM45 protein frequently exists in a high-molecular weight, oligomeric conformation, presumably as part of its normal function(s) [4, 7]. This type of RBP self-association or oligomerization is often required for or accompanies RBP incorporation into membraneless organelles such as SGs, P-bodies, nucleoli, paraspeckles, and Cajal bodies [8, 9]. We also used immunoprecipitation coupled to mass-spectrometry (IP-MS) experiments to identify RBM45-interacting proteins and infer that RBM45 regulates mRNA splicing and spliceosome function in the nucleus [3]. Collectively, our observations that RBM45 self-associates and regulates mRNA processing suggest that the protein may be a component of one or more membraneless nuclear organelles that regulate mRNA splicing and processing, such as nuclear speckles or Cajal bodies.

While self-association of RBPs is critical for membraneless organelle formation and function, this process also contributes to inclusion formation in diseases such as ALS and FTLD [8, 10–13]. A well-studied example of this phenomenon is the RBP aggregation and inclusions that result from RBP incorporation and entrapment in cytoplasmic SGs [14, 15]. SGs are cytoplasmic protein-mRNA complexes that form in response to cellular stress [16, 17]. They protect mRNAs from a harmful cellular environment and stall their translation, thus conserving energy and prioritizing translation of stress response-associated proteins (reviewed in [16, 17]). TDP-43 and FUS are components of SGs and chronic confinement of these RBPs in SGs results in the formation of insoluble inclusions containing these proteins in a variety of model systems [14, 18–21]. Likewise, SG marker proteins are found in TDP-43 and FUS-positive neuronal cytoplasmic inclusions in ALS and FTLD patients, suggesting that SGs are capable of seeding inclusion formation [19, 22]. The presence of low complexity and intrinsically disordered domains in these and other SG-associated RBPs facilitates aggregation and can lead to the formation of several forms of amyloid or amyloid-like structures, including amyloidogenic oligomers and fibrils [23–26]. This pathological “maturation” of RBP assemblies into insoluble aggregates and inclusions removes RBPs from their normal cellular milieu, leading to loss of normal RBP function(s) [27–30]. We propose that an analogous process occurring in the nucleus contributes to both the normal functions of RBM45 and the formation of the nuclear RBM45 inclusions seen in ALS, FTLD-TDP, and AD.

To test this hypothesis, we explored the association of RBM45 with a variety of membraneless nuclear organelles, including nuclear speckles, Cajal bodies, nuclear gems, and nuclear stress bodies (NSBs). We found that RBM45 is recruited to NSBs following exposure to a diverse array of cellular stressors. This association is dependent on the protein’s NLS and ability to bind RNA. The persistent association of RBM45 with NSBs is sufficient to promote its aggregation into insoluble inclusions and, unlike NSBs, these complexes do not disassemble, but instead increase in size and number over time. Collectively, our results (1) identify novel functions of RBM45; (2) define a new mechanism by which the stress-associated functions of RBPs contribute to their aggregation and inclusion formation; and (3) provide quantitative measures of the cell type and disease-specific patterns of RBM45 pathology that occur in ALS, FTLD, and AD.

## Methods

### Cell culture, transfection, and nuclear stress body induction

HEK293 cells or FreeStyle 293F cells (Invitrogen, Waltham, MA, USA; RRID:CVCL_0045 and RRID:CVCL_D603, respectively) were cultured in DMEM medium with 10% FBS and 1% Pen-Strep at 37 °C with 5% CO_2_. Mycoplasma detection was performed using DAPI staining and the Lookout Mycoplasma PCR detection kit (Sigma-Aldrich, St. Louis, MA, USA). The Lipofectamine 3000 reagent (Invitrogen) was used to transfect cells according to the manufacturer’s protocol and transfected cells were harvested 48 or 72 h post-transfection. Tet-inducible EGFP-RBM45 stable cell lines (see below) were maintained in the presence of 50 μg/ml Zeocin (InvivoGen, San Diego, CA, USA). EGFP-RBM45 expression was induced by adding 1 μg/ml doxycycline hyclate (Sigma-Aldrich) for 16 h before live-cell imaging.

To induce nuclear stress body (NSB) formation, we used a variety of previously described stressor protocols. Heat shock was performed by incubating the cells in a 42 °C environment for various durations (ranging from 0.5 to 2 h, as indicated in the text/figures) followed by various recovery times, ranging from 1 to 24 h, as indicated [31, 32]. The following reagents were added to culture medium at the indicated concentrations to induce NSB formation, as previously described: 5–30 μM CDSO_4_ (2–24 h) [32, 33], 200–400 μM H_2_O_2_ (2–24 h) [32, 34], 0.1–1 mM sodium arsenite (0.25–24 h) [35], or 0.5–20 μM mitoxantrone (MTX; 2–24 h) [36].

### Plasmid construction

For expression of WT and domain deletion RBM45 constructs, a plasmid containing RBM45, cGST-hRBM45 (HsCD00356971), was obtained from the DNASU Plasmid Repository at Arizona State University, Tempe, AZ, USA. The RBM45 cDNA was amplified by PCR using Phusion High-Fidelity DNA Polymerase (NEB) and sub-cloned into the pcDNA3 vector (Invitrogen). A 3xFLAG tag (DYKDHDGDYKDHDIDYKDDDDK) or 2xHA tag (DYPYDVPDYAGGAAYPYDVPDYA) was appended to the N-terminus of RBM45 to generate the 3xFLAG- or 2xHA-tagged constructs, respectively. In previous experiments, we found that appending tags to the C-terminus of the protein alters its subcellular localization, presumably by interfering with the function of the C-terminal nuclear localization sequence (amino acids 454–472 of 474). Thus, all RBM45 tags were appended to the protein’s N-terminus. Domain deletions and mutations were introduced by site-directed mutagenesis using overlap extension PCR [37]. Sequences of RBM45 domain deletion and non-functional NLS mutant constructs were as in Li et al. [4]. For live-cell imaging of RBM45 dynamics during conditions of cellular stress, a Tet-inducible EGFP-RBM45 construct was generated by inserting the EGFP-RBM45 (N-terminal EGFP fusion) into the pcDNA4/TO vector (Invitrogen). The sequences of all constructs were confirmed by Sanger DNA sequencing and the sizes of the expressed proteins confirmed by Western blot.

### Inducible EGFP-RBM45 cell line

#### Creation of the Tet-on parental stable cell line

HEK293 cells were transfected with pcDNA6/TR (Invitrogen) to express the Tet repressor and selected with 10 μg/ml Blasticidin (InvivoGen, San Diego, CA, USA). Multiple stable clones were isolated, propagated, transfected with pcDNA4/TO/lacZ (Invitrogen), and induced with 10 μg/ml doxycycline hyclate (Sigma-Aldrich) for the expression of ß-galactosidase. The ß-Gal assay was performed to screen for clones with the highest expression of the Tet repressor. A Tet-on parental clone with a combination of the lowest ß-galactosidase levels without doxycycline induction and highest expression of ß-galactosidase in the presence of doxycycline was then selected. This line was maintained with 5 μg/ml Blasticidin.

#### Creation of inducible EGFP-RBM45 cell line

pcDNA4/TO-EGFP-RBM45 was transfected into the Tet-on parental stable cell line above and EGFP-positive stable cell lines were selected in the presence of 100 μg/ml Zeocin.

### CRISPR-Cas9 modified FLAG-RBM45 cell line

A 3xFLAG tag was appended to the N-terminus of the endogenous RBM45 genomic locus in HEK293 cells using the CRISPR-Cas9 method described by Ran et al. [38] with some modifications.

#### sgRNA preparation

Five sgRNA sequences near the N-terminus of the RBM45 genomic locus were selected using the CRISPR Design Tool (http://crispr.mit.edu) [38]. Oligos encoding the five sgRNAs were individually cloned into the pSpCas9(BB)-2A-Puro (PX459) V2.0 vector (RRID:Addgene_62988) and transfected into HEK293 cells. Cells were selected in the presence of 5 μg/ml puromycin (InvivoGen) for 48 h before functional validation of sgRNAs. The cleavage efficiency of each sgRNA was evaluated by the TIDE (Tracking of Indels by DEcomposition) assay [39]. Briefly, genomic DNA from puromycin selected cells and wild-type HEK293 cells was extracted (Promega Wizard Genomic DNA Purification Kit #A1120; Promega, Madison, WI, USA) for PCR amplification of a 535 bp region flanking the sgRNA cleavage site. The PCR DNAs were gel purified (Promega Wizard SV Gel and PCR Clean-up System #A9282) and 30 ng of each PCR DNA was Sanger sequenced. The sequencing result for each sgRNA was aligned to the wild-type control by TIDE software (https://tide.nki.nl/) and the resulting traces were used to calculate cleavage efficiencies [39]. The two sgRNAs with the highest cleavage efficiency (both 85%) were chosen for co-transfection with a homology-directed repair (HDR) template for CRISPR-Cas9 genome editing.

#### Generation of CRISPR-Cas9 modified FLAG-RBM45 HEK293 cell line

A 200 nt ssODN ultramer (IDT) was used as a homology-directed repair (HDR) template for CRISPR-Cas9 editing of the endogenous RBM45 locus. Co-transfection of 500 ng sgRNA plasmid and 1 μl of 10 μM ssODN template into 2 × 10^5^ HEK293 cells was performed using nucleofection on the 4D-Nucleofector system (LONZA, Basel, Switzerland), as in [38]. The transfected cells were enriched by selection with 1 μg/ml puromycin for 48 h. Clonal cell lines were isolated by serial dilution and expanded for 3 weeks. Genomic DNA from cell lines growing on 96-well plates was extracted by 25 μl QuickExtract DNA extraction solution (Epicentre #QE09050, Madison, WI, USA), further diluted to 200 μl with H_2_O. CRISPR-Cas9 genome editing was assessed by using genomic DNA from these samples for PCR amplification of the RBM45 genomic locus using a FLAG-specific forward primer and an RBM45 reverse primer flanking a 480 bp region. The 480 bp PCR DNAs were then Sanger sequenced to verify the correct insertion of the 3xFLAG into the desired genomic locus. Real-time PCR was used to measure RBM45 mRNA levels in wild-type and FLAG-RBM45 expressing cell lines. We then used immunostaining to assess the nuclear morphology, subcellular localization, and RBM45 protein levels in CRISPR modified FLAG-RBM45 and wild-type HEK293 cells.

#### Primers used for TIDE assay

PCR forward primer: 5′-GGACTCCTCTTTCTCCCGGAAGCGG

PCR reverse primer: 5′-GCTGCCGGGAACGGATGTTCCACTC

Sequencing forward primer: 5′-ACTCCTCTTTCTCCCGGAAGCGGAG

Sequencing reverse primer: 5′-TGCCGGGAACGGATGTTCCACTCCT

#### Target sequences of the two sgRNAs with the highest cleavage efficiency

5′-TGCATTCGGGTGGAGCACCA (sense strand)

5′-AGAGCTGCCAGCTTCGTCCA (anti-sense strand)

#### Sequence of ssODN

Uppercase letters indicate the sequence for the 2xFLAG tag:

5′-gagacagcagcggtggcagacaccgcagaagcaaagagcagtgaggctcctgcattcgggtggagcaccatgGACTACAAAGACGATGACGACAAGGACTACAAGGATGACGATGACAAAGCTGCTgacgaagctggcagctctgcgagcggcgggggcttccgcccgggcgtggacagcctggacgaaccgcccaaca

#### Primers to PCR amplify CRISPR modified endogenous FLAG-RBM45

FLAG-specific forward primer: 5′-TCGGGTGGAGCACCATGGACTACAAAG

RBM45 reverse primer: 5′-GCTGCCGGGAACGGATGTTCCACTC

### siRNA knockdown of RBM45 and SatIII

To assess the effects of knockdown of endogenous RBM45 or SatIII expression on NSB formation, siRNAs targeting each transcript were designed based on previous studies [40, 41] and transfected into HEK293 cells. Two siRNAs targeting RBM45 and one siRNA targeting SatIII were used, along with a scrambled siRNA as a negative control. For all siRNA knockdown studies, 40% confluent HEK293 cells growing on 24- or 96-well plates were transfected with 15 pmol or 3 pmol of siRNA, respectively, using the Lipofectamine 3000 reagent (Invitrogen) according to the manufacturer’s instructions. Cells were immunostained or assayed for cellular viability 48 h post-transfection. siRNAs (Dharmacon, Layfayette, CO, USA) with the following target sequences were used:

RBM45–1: 5′-GUAUGGAGAUAUCGAGUAU

RBM45–2: 5′-GGACAUGAACCUAGAGUAA

SatIII: 5′-UGGAAUGGAAUGGAAUGGA

The negative control scrambled siRNA (Dharmacon # D-001810-10-05) consists of a pool of 4 sequences previously determined to have minimal effects on gene expression in mammalian cells [42]. Real-time PCR was used to quantify the siRNA knockdown efficiency for each transcript. cDNAs were made with SuperScript VILO Mastermix (Invitrogen) containing the random hexamer and OligodT primers. The relative quantity was normalized with GAPDH and calculated using the ∆∆Ct method. The primers for real-time PCR were:

RBM45: fwd = 5′-CCTTATTCAAATTATGGTCATGGAG

rev = 5′-GTGTTGCCATTTTTCTAAGGAGATCTG

SatIII: fwd = 5′-TATGAATTCAATCAACCCGAGTGCAATCGAA

rev = 5′-TATGGATCCTTCCATTCCATTCCTGTACTCG

GAPDH: fwd = 5′-GAAATCCCATCACCATCTTCCAGG

rev = 5′-GAGCCCCAGCCTTCTCCATG

### Subcellular fractionation and assessment of RBM45 solubility

HEK293 cells were grown in 10 cm tissue culture dishes for fractionation into nuclear/cytoplasmic or total protein extracts. All extraction procedures were done at 4 °C in the presence of a protease inhibitor cocktail (Sigma-Aldrich). Cytoplasmic extracts were obtained by immersing cells in a hypotonic buffer (10 mM HEPES, 1.5 mM MgCl_2_, 10 mM KCl, 0.5 mM DTT) for 5 min and then lysing cells by homogenization with a Dounce homogenizer. The lysed cells were spun for 5 min at 1000 rpm to pellet nuclei and the supernatant was retained as the cytoplasmic fraction and solubilized in RIPA buffer. Cells were visualized by brightfield microscopy to confirm hypotonic buffer-induced swelling, cell membrane lysis, and nuclear membrane integrity following homogenization. The pelleted nuclei were washed, re-suspended, centrifuged at 2800 g for 10 min, and broken with sonication, which was also monitored by brightfield microscopy. Following centrifugation at 2800 g for 10 min, the supernatant was retained as the nuclear fraction and solubilized in RIPA buffer. The purity of the lysate was determined by SDS-PAGE and Western blotting for GAPDH and lamin A/C as cytoplasmic and nuclear markers, respectively.

For assays examining RBM45 solubility under various stressor conditions, HEK293 cells constitutively expressing FLAG-RBM45 (overexpressed or CRISPR-modified, as described above) were grown on 10 cm plates until confluent and were treated as indicated to induce the formation of NSBs. The cells were harvested and fractionated into soluble and insoluble protein fractions as previously described [43] with minor modifications. Cells from one 10 cm plate were harvested, lysed in 1.5 ml RIPA buffer (150 mM NaCl, 1% NP-40, 0.1% SDS, 50 mM Tris, pH 8.0) at 4 °C for 15 min, sonicated, and centrifuged at 100,000 g for 30 min at 4 °C. After centrifugation, the supernatant was collected as the RIPA-soluble fraction and its protein concentration was determined by BCA assay (Pierce). To prevent carryover of residual soluble protein extract into the insoluble fraction, the resulting pellets were washed once with RIPA buffer, resonicated, recentrifuged at 100,000 g for 30 min at 4 °C, and the supernatant was removed. RIPA-insoluble pellets were then extracted in 1.5 ml urea buffer (7 M urea, 2 M thiourea, 4% CHAPS, 30 mM Tris, pH 8.5) at room temperature for 20 min, sonicated, and centrifuged at 100,000 g for 30 min at 22 °C. The final supernatant was collected as the insoluble, detergent-resistant fraction and its protein concentration was determined by Bradford assay (Bio-Rad, Hercules, CA, USA). To inhibit protein degradation, 1 mM PMSF (a protease inhibitor) was added to all buffers prior to use.

### SDS-PAGE and Western blotting

SDS-PAGE, total protein staining, and Western blotting were performed as previously described [44]. In brief, samples were mixed with LDS sample buffer (Invitrogen), DTT reducing reagent, and deionized water (as a diluent), heated at 70 °C for 10 min, and loaded onto 1.5 mm 4–12% Bis-Tris gradient SDS-PAGE gels (Invitrogen). For assays examining HSF1, SAFB, and RBM45 abundance, 10 μg of protein was loaded from lysates prepared as above. For assays examining the solubility of RBM45, 15 μg of the RIPA-soluble fraction and 4 μg of the detergent-resistant fraction were loaded in each lane. Gel runs were performed in 1X MOPS buffer at a constant 200 V at 4 °C. Following SDS-PAGE, proteins were transferred from gels to PVDF membranes. Wet tank transfer was performed in Towbin buffer (25 mM Tris, 192 mM glycine) using a ramped transfer approach, as in Otter et al. [45], where samples were transferred for 6 h at a constant 8 V, then for a further 6 h at 16 V. Following the transfer, membranes were dried for 1 h, then rehydrated using methanol and deionized water.

For Western blotting, membranes were initially blocked in Odyssey blocking buffer (Licor) for 60 min. Membranes were incubated in primary antibodies for 1–12 h and washed three times for 15 min each in a phosphate buffered saline (PBS) solution with Odyssey blocking buffer added at a dilution of 1:10. Following the washing step, membranes were incubated in solutions containing secondary antibodies conjugated to IRDye 680 and IRDye 800 (LI-COR, Lincoln, NE, USA) for 60 min and washed as above. After secondary antibody incubations membranes were washed 3 times for 15 min each in Odyssey blocking buffer diluted 1:10 in PBS. Finally, immediately prior to imaging, membranes were washed once in PBS to remove residual detergent from the membrane. Blot images were acquired on an Odyssey CLx scanner (LI-COR) at 169 μm resolution at the highest intensity setting at which no pixel saturation was present in the resulting image.

### Immunocytochemistry and Digital Deconvolution

Indirect immunofluorescence was performed as in Li et al. [3] In brief, cells were grown on 20 mM #1.5 poly-D-lysine (PDL)-coated coverslips. When cells reached 70% confluence, they were treated as indicated, washed with 1X PBS, fixed in 4% paraformaldehyde for 10 min, washed 3 times in PBS, and permeabilized for 10 min using 0.1% Triton X-100 in PBS. Following additional washing with 1X PBS to remove fixative and detergent, cells were blocked with Superblock (Scytek, Logan, UT, USA) for 1 h and immersed in primary antibody solutions for 2 h. Subsequently, they were washed 4 times (10 min each) with IF wash buffer (1:10 Superblock:1X PBS), and immersed in secondary antibody solutions for 1 h. The secondary antibodies used were goat-anti-rabbit Cy2 (Abcam, Cambridge, UK) and goat-anti-mouse Cy5 (Abcam). Following secondary antibody incubations, coverslips were washed 4 times as above, washed 4 times with 1X PBS, incubated in a 300 nM DAPI solution for 10 min, and washed 4 times with 1X PBS. Coverslips were immersed in increasing concentrations of 2,2′-thiodiethanol (TDE) according to the method of Staudt et al. [46], and mounted in a 97% TDE solution with a refractive index of 1.518 to match that of the immersion oil used for imaging.

Slides were imaged on an Observer Z1 (Zeiss, Jena, Germany) microscope with a 1.4 NA 63x objective with LED illumination. All images were acquired as three-dimensional Z-stacks with a Z sampling depth of 12 μM, X/Y sampling interval of 0.102 μM, and a Z sampling interval of 0.240 μM. We performed shading correction and background subtraction on all images prior to deconvolution. Following image acquisition and initial processing, images were deconvolved using Huygens digital deconvolution software (SVI; RRID:SCR_014237). A measured point spread function (PSF) was generated by imaging 200 nm diameter fluorescent Tetraspeck beads (a sub-resolution particle in this imaging system) (Life Technologies, Waltham, MA, USA) mounted in 97% TDE and inputting the obtained images into the Huygens software’s PSF distiller application. The performance of the measured PSF was assessed by deconvolving additional 10 μm image stacks of 200 nm fluorescent beads using the measured and theoretical PSFs. Comparisons of the deconvolution result using the different PSFs demonstrated that the measured PSF provided a superior image restoration result compared to a theoretical PSF, as assessed by the size and shape of the deconvolved bead image. Alignment of individual channels in the measured PSF was then used to correct images for spherical aberration. All cell images were then deconvolved using the measured PSF and a maximum likelihood deconvolution algorithm.

### Immunohistochemistry

Immunohistochemistry of lumbar spinal cord and hippocampal brain tissue sections from non-neurological disease control, sporadic ALS (sALS), c9ORF72 hexanucleotide repeat expansion ALS (c9ORF72), and non-c9ORF72 linked familial ALS (fALS), frontotemporal lobar degeneration with TDP-43 proteinopathy (FTLD-TDP), and Alzheimer’s disease (AD) subjects (all AD subjects were Braak stage VI) was performed as previously described [1]. Tissue sections were obtained from the Barrow Neurological Institute and Target ALS Multicenter post-mortem tissue bank cores. All participants in the post mortem tissue bank cores provided IRB approved informed consent for the collection of post mortem tissues. Subject demographics are shown in Table 1. Formalin fixed, paraffin embedded tissue sections (6 μm thick) were deparaffinized by immersion in xylene, rehydrated by successive immersion in increasing concentrations of ultrapure water in ethanol, and subjected to antigen retrieval. Antigen retrieval was performed in citra buffer (pH 6.0; Biogenex, Fremont, CA, USA) by steam heating for 30 min. Sections were washed in PBS, blocked using Superblock (Scytek), and immersed in primary antibody solutions overnight. The next day, the slides were washed 4 times in PBS and incubated in solutions containing appropriate secondary antibodies conjugated to Alexafluor 488 and 594 for 1 h. Subsequently, slides were again washed 4 times in PBS, and immersed in a 300 nM DAPI solution for 10 min to stain cell nuclei. Following PBS washing, slides were immersed in a Sudan black-based autofluorescence eliminator reagent (Millipore) to quench endogenous lipofuscin autofluorescence and mounted in gelvatol. All tissue immunofluorescence images were acquired on a Zeiss LSM 720 confocal microscope. Images were acquired as three-dimensional Z-stacks with a Z sampling range of 15 μm, X/Y sampling interval of 0.102 μM, and a Z sampling interval of 0.240 μm. Shading correction and background subtraction were performed on all images.

**Table 1:** Subject demographics. Demographics for each case used for immunohistochemical analysis are shown. PMI = post mortem interval (hours between death and tissue harvest); C9orf72 = presence or absence of C9orf72 hexanucleotide repeat expansion; sALS = sporadic (i.e., non-familial) ALS; AD = Alzheimer’s disease, Braak stage VI; FTLD-TDP = frontotemporal lobar degeneration with TDP-43 proteinopathy

Subject	Group	C9orf72	Age	Sex	PMI	Hippocampus	Spinal Cord
1	Control	no	54	M	6	yes	yes
2	Control	no	53	F	4	yes	yes
3	Control	no	51	F	5	yes	yes
4	Control	no	76	M	13	yes	yes
5	Control	no	57	M	2	yes	yes
6	Control	no	48	M	2	yes	yes
7	Control	no	58	F	5	yes	yes
8	Control	no	76	M	14	yes	no
9	Control	no	82	F	5	yes	yes
10	Control	no	57	F	11	yes	yes
11	sALS	no	62	M	7	no	yes
12	sALS	no	50	F	7	no	yes
13	sALS	no	60	F	4	no	yes
14	sALS	no	59	M	4	yes	yes
15	sALS	no	77	F	4	yes	yes
16	sALS	no	79	F	4	yes	yes
17	sALS	no	52	F	21	yes	yes
18	sALS	no	45	M	7	yes	no
19	sALS	no	65	M	5	yes	no
20	sALS	no	66	M	18	yes	yes
21	sALS	no	53	F	12	yes	yes
22	sALS	no	73	F	6	no	yes
23	sALS	no	59	M	5	no	yes
24	sALS	no	48	F	5	no	yes
25	ALS	yes	68	M	4	no	yes
26	ALS	yes	43	M	6	no	yes
27	ALS	yes	63	F	4	no	yes
28	AD	no	71	M	4	yes	no
29	AD	no	84	F	5	yes	no
30	AD	no	85	M	5	yes	no
31	AD	no	73	F	4	yes	no
32	AD	no	71	F	5	yes	no
33	FTLD-TDP	no	69	M	6	yes	no
34	FTLD-TDP	no	67	M	> 24	yes	no
35	FTLD-TDP	no	69	M	6	yes	no
36	FTLD-TDP	no	85	M	3	yes	no
37	FTLD-TDP	no	91	F	8	yes	no
38	FTLD-TDP	no	79	M	7	yes	no

### Image analysis

Image analysis was performed using NIH ImageJ (RRID:SCR_003070) [47]. We developed an image analysis pipeline to (1) count the number of cells in an image field, (2) count the number of RBM45 nuclear inclusions in each cell in an image field, and (3) measure the nuclear fluorescence intensity in each fluorescence channel in each cell in each image field. These steps were carried out as follows. To count the number of cells in each image, we created binary images of DAPI-stained nuclei using fixed intensity thresholding. The ImageJ particle analyzer was then used to count the number of DAPI-positive cells in each image. Cell counts were further filtered by excluding possible false-positives on the basis of cell size and shape. To count the number of RBM45 nuclear inclusions, we used the DAPI binary images as an overlay mask to restrict analysis to RBM45 foci in the nuclei of cells. These masked images were then thresholded using the Robust Automatic Threshold plugin [48]. The ImageJ particle analyzer was then used to count the number of RBM45-positive foci in the nucleus with particle sizes restricted to published parameters for the size of nuclear stress bodies [33, 49]. Finally, the intensity of each fluorescence channel in each cell nucleus in an image field was measured using the corresponding DAPI binary image as an overlay mask. We applied this image analysis pipeline to cells from our immunocytochemistry experiments, hippocampal dentate gyrus granule cells from ALS, FTLD-TDP, AD, and non-neurological disease control tissue sections, and spinal cord motor neurons and glia from ALS and non-neurological disease control tissue sections. For each subject from our human tissue analysis, a total of 8 fields per region (hippocampal dentate gyrus or lumbar spinal cord) were analyzed. Each step in the image analysis pipeline was evaluated by comparing its performance to manual counts of at least 10 independent images, with a requirement of 95% agreement between automated and manual counts across images.

### Antibodies

The following primary antibodies and dilutions were used for all Western blotting experiments, rabbit polyclonal anti-C-terminal RBM45 (Pacific Immunology, Fremont, CA, USA, custom, 1:2000), rabbit polyclonal anti-N-terminal RBM45 (Pacific Immunology, custom, 1:2000), rabbit polyclonal anti-RBM45 (Sigma-Aldrich, 1:1000; RRID:AB_1856132), mouse-monoclonal anti-HA (Sigma-Aldrich, 1:5000; RRID:AB_260092), mouse monoclonal anti-FLAG M2 (Sigma-Aldrich F3165, 1:5000; RRID:AB_259529), rabbit polyclonal anti-TDP-43 (Proteintech, Rosemont, IL, USA, 1:3000; RRID:AB_2200505), mouse monoclonal anti-actin (Millipore, Burlington, MA, USA, 1:5000; RRID:AB_2223041), rabbit monoclonal anti-lamin A/C (Epitomics, San Francisco, CA, USA, 1:1000; RRID:AB_10860619), and rabbit monoclonal anti-GAPDH (Cell Signaling, Danvers, MA, USA, 2118S, 1:5000; RRID:AB_561053). The following secondary antibodies were used for Western blotting experiments, each at a dilution of 1:10,000: goat anti-rabbit IRDye 680 (LI-COR; RRID:AB_10956166), goat anti-rabbit IRDye 800 (LI-COR; RRID:AB_621843), goat anti-mouse IRDye 680 (LI-COR; RRID:AB_10956588), and goat anti-mouse IRDye 800 (LI-COR; RRID:AB_621842).

The following primary antibodies and dilutions were used for immunofluorescence and immunohistochemistry: rabbit polyclonal anti-RBM45 (Sigma-Aldrich, 1:75; RRID:AB_1856133), rabbit polyclonal anti-RBM45 (custom; Pacific Immunology, 1:100), mouse monoclonal anti- scaffold attachment factor B (SAFB, Lifespan Biosciences, Seattle, WA, USA, 1:100; RRID:AB_2183072), mouse monoclonal anti-SAFB (Proteintech, 1:100), rabbit polyclonal anti-SAFB (Proteintech, 1:100), mouse-anti-HSF1 (Abcam, 1:100), rabbit polyclonal anti-heat shock factor 1 (HSF1; Proteintech, 1:100; RRID:AB_10903615), mouse monoclonal anti-SC35 (Abcam, 1:300; RRID:AB_298608), mouse monoclonal anti-coilin (Abcam, 1:200; RRID:AB_476827), mouse monoclonal anti-SMN (Sigma-Aldrich, 1:300; RRID:AB_477504), mouse monoclonal anti-G3BP1 (Genentech, 1:100), mouse monoclonal anti-TDP-43 (Proteintech, 1:100; RRID:AB_2200520), mouse monoclonal anti-FUS (Proteintech, 1:100; RRID:AB_10666169), and mouse monoclonal anti-HA (Sigma-Aldrich, 1:1000; RRID:AB_260092). Goat-anti-rabbit Cy2 (Abcam; RRID:AB_954992) and goat-anti-mouse Cy5 (Abcam; RRID:AB_955063) secondary antibodies were used for immunofluorescence at a dilution of 1:200. Goat-anti-rabbit Alexafluor 488 (Life Technologoies; RRID:AB_2576217) and goat-anti-mouse Alexafluor 594 (Life Technologies; RRID:AB_2534091) secondary antibodies were used at a dilution of 1:200 for human tissue immunohistochemistry experiments.

### Data analysis

The statistical analysis of all data was performed using Microsoft Excel (Microsoft, Redmond, WA, USA; RRID:SCR_016137) and R (R Project for Statistical Computing; RRDI:SCR_001905). Between groups comparisons were performed using one-way analysis of variance (ANOVA) with Tukey’s HSD post-hoc test. For the analysis of RBM45 nuclear inclusions per cell in human tissues, we used the non-parametric Mann-Whitney U test to perform all possible two group comparisons. The Bonferroni correction was used to account for multiple testing in these comparisons. Linear regression was used to analyze the relationship between RBM45 nuclear inclusion number and SAFB nuclear immunoreactivity. All figures were constructed in Adobe Illustrator (Adobe, Mountain View, CA, USA; RRID:SCR_010279) and Inkscape (RRID:SCR_014479).

## Results

### RBM45 associates with nuclear stress bodies (NSBs)

**Fig. 1:**
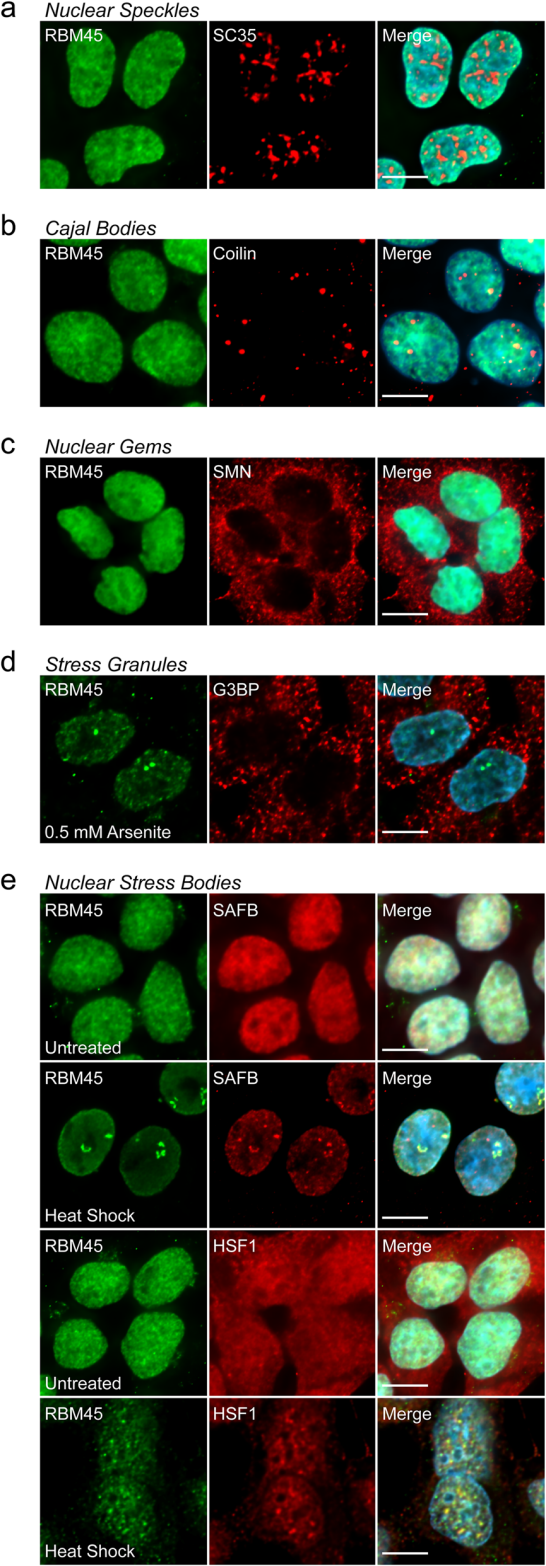
RBM45 association with membraneless nuclear organelles. **a-c** HEK293 cells were immunostained for endogenous RBM45 and marker proteins of the indicated organelles. Under basal conditions, RBM45 is diffusely localized throughout the nucleus and does not associate with nuclear speckles, Cajal bodies, or nuclear gems (**a-c**, respectively). **d** Sodium arsenite (0.5 mM for 1 h) was used to induce cytoplasmic stress granule formation. RBM45 does not associate with G3BP1-positive cytoplasmic stress granules during cellular stress, but coalesces into nuclear puncta. **e** Heat shock (42 °C for 1 h) led to a redistribution of SAFB (top) and HSF1 (bottom) into nuclear stress bodies (NSBs). Stress-induced RBM45 nuclear foci correspond to NSBs, indicated by co-localization of RBM45 with the NSBs. For all images, scale bar = 5 μm

To better understand the normal functions of RBM45 and the mechanisms leading to nuclear RBM45 inclusion formation, we assessed RBM45’s co-localization with markers of several membraneless organelles in HEK293 cells. We used established marker proteins for nuclear speckles (SC35), Cajal bodies (coilin), nuclear gems (SMN), cytoplasmic stress granules (SGs; G3BP), and nuclear stress bodies (NSBs; SAFB and HSF1). As shown in Fig. 1a-c, under basal conditions RBM45 is a predominantly nuclear protein with minimal cytoplasmic immunoreactivity. Moreover, the protein is diffusely localized throughout the nucleus and does not co-localize with nuclear speckles, Cajal bodies, or nuclear gems.

We next examined whether cellular stress promotes redistribution of RBM45 into stress-induced membraneless organelles. Sodium arsenite (0.5 mM for 1 h) led to robust induction of G3BP-positive cytoplasmic stress granules (Fig. 1d), however, cytoplasmic SGs did not contain RBM45. Instead, sodium arsenite caused the diffusely localized RBM45 to coalesce into nuclear puncta (Fig. 1d). We then used heat shock (42 °C for 1 h) to induce redistribution of SAFB and HSF1 into NSBs (Fig. 1e). As with sodium arsenite, we found that heat shock led to the formation of nuclear RBM45 puncta and these puncta co-localized with SAFB- and HSF1-positive NSBs (Fig. 1e).

**Fig. 2:**
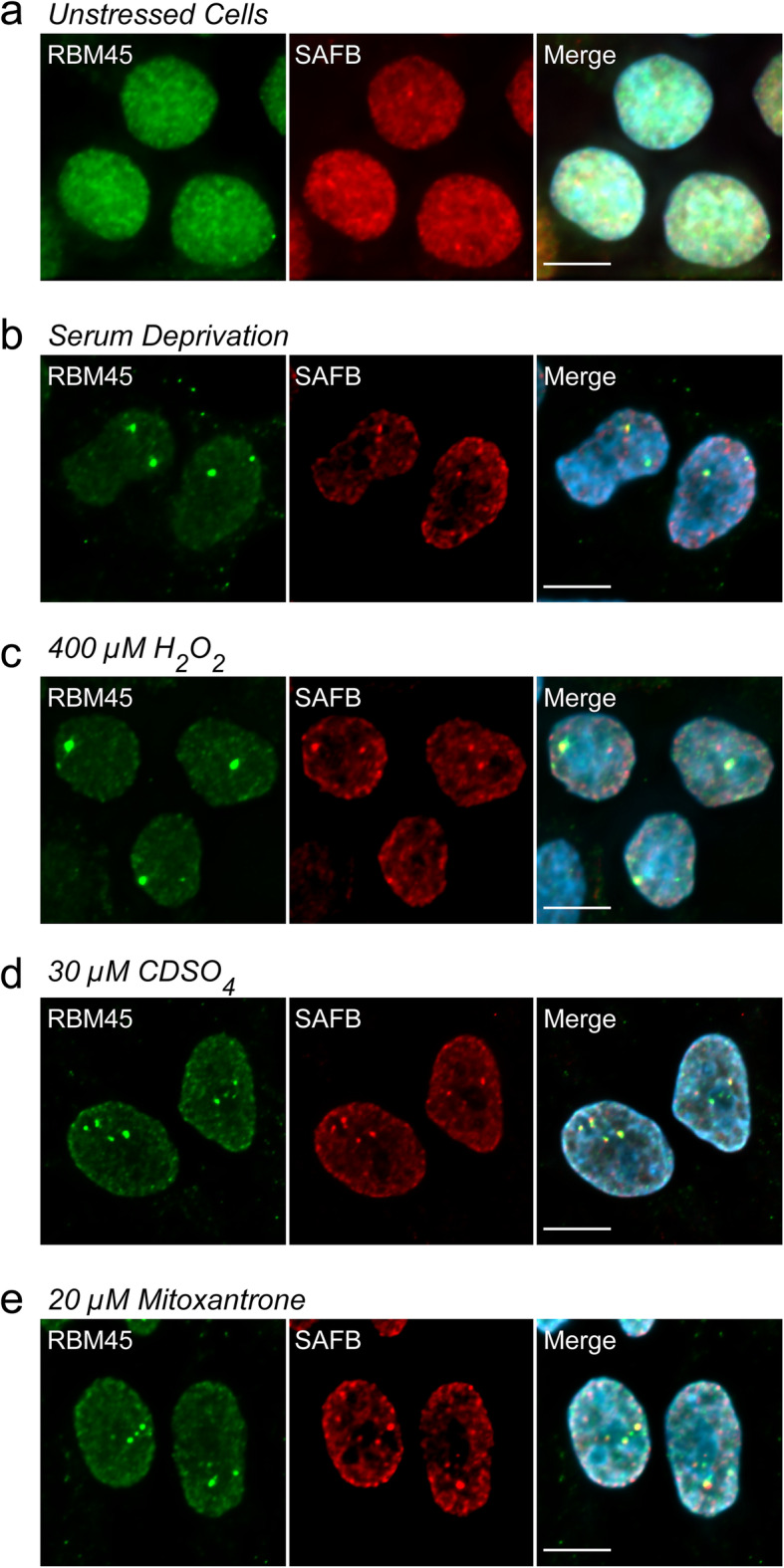
RBM45 association with nuclear stress bodies is a general response to cellular stress. HEK293 cells were treated as indicated and the distribution of RBM45 and SAFB-positive nuclear stress bodies (NSBs) was evaluated by immunocytochemistry. **a** In untreated cells, the distribution of RBM45 and SAFB is diffuse and nuclear. **b-e** Treatment with the indicated stressors results in the formation of RBM45- and SAFB-positive NSBs. For all images, scale bar = 5 μm

Because both sodium arsenite and heat shock led to the formation of RBM45-positive NSBs, we asked whether RBM45 incorporation into NSBs is a general response to cellular stress. In addition to sodium arsenite and heat shock (Fig. 1e), we found that other NSB-inducing stressors, including nutrient starvation (serum deprivation, Fig. 2b), oxidative stress (400 μM H_2_O_2_, Fig. 2c), heavy metal stress (30 μM cadmium sulfate [CdSO_4_], Fig. 2d), and genotoxic stress (20 μM mitoxantrone, Fig. 2e) led to the formation of RBM45-positive NSBs. As in our initial experiments, under basal conditions, the distribution of RBM45 was diffuse and nuclear, with no RBM45 puncta or NSBs visible (Fig. 2a). The stress conditions used in these experiments also cause the formation of cytoplasmic SGs [35, 50–55]. Because previous reports indicated that RBM45 associates with cytoplasmic SGs [4, 5], we also examined whether any of the stressors tested led to the incorporation of RBM45 into SGs. We found that all stressors led to the formation of G3BP-positive SGs, but these did not contain RBM45 (Fig. S1).

NSBs are protein-RNA complexes that result from stress-induced transcription of pericentromeric heterochromatic satellite III (SatIII) repeats. SatIII transcripts remain in close proximity to their genomic locus and act as scaffolds for NSB assembly by recruiting various NSB RNA binding proteins, including HSF1 and SAFB. NSBs are proposed to result from a redistribution of existing nuclear RBPs, rather than an increase in NSB protein levels. To assess whether cellular stress alters RBM45 protein levels, we performed Western blotting for RBM45 in HEK293 total protein extracts from unstressed HEK293 cells and cells subjected to oxidative or genotoxic stress. Neither stressor increased RBM45, HSF1, or SAFB protein levels in HEK293 cells (*p* > 0.05; Fig. S2), consistent with existing models of NSB formation [31, 49, 56, 57].

We examined if RBM45-interacting proteins linked to ALS are recruited to NSBs by virtue of their interactions with RBM45. TDP-43 and FUS physically interact with RBM45 [4], are predominantly nuclear proteins under basal conditions, and have defined roles in the cellular response to stress. We therefore assessed whether TDP-43 or FUS can be incorporated into NSBs during cellular stress. Heat shock did not lead to the incorporation of either TDP-43 or FUS into RBM45-positive NSBs (Fig. S3A). We overexpressed HA-tagged versions of full-length TDP-43 and FUS and evaluated their effects on NSB formation and their association with NSBs. As in the preceding experiments, we did not observe association of TDP-43 or FUS with NSBs (Fig. S3B). Overexpression of TDP-43, but not FUS, was sufficient to promote NSB formation, consistent with previous studies showing that altered TDP-43 expression induces cellular stress (Fig. S3B) [56, 57].

We also tested whether RBM45 is required for the formation of NSBs using siRNA-based knockdown of RBM45. qPCR experiments established that our RBM45 siRNAs effectively targeted RBM45, resulting in an approximately 70% decrease in transcript abundance (Fig. S4). As a positive control for these experiments, we used siRNAs targeting SatIII, as prior studies demonstrated that SatIII knockdown abrogates NSB formation [40, 41]. SatIII siRNAs reduced transcript levels approximately 80% (Fig. S4). Neither the scrambled siRNA nor knockdown of RBM45 prevented the formation of NSBs (Fig. S5A, B, respectively). Consistent with previous findings, knockdown of SatIII transcripts prevented NSB formation and eliminated redistribution of RBM45, SAFB, and HSF1 to nuclear foci during cellular stress (Fig. S5C). Therefore, RBM45 associates with NSBs during conditions of cellular stress, but is not essential for NSB formation.

### RNA binding domains are essential for RBM45 association with NSBs

**Fig. 3:**
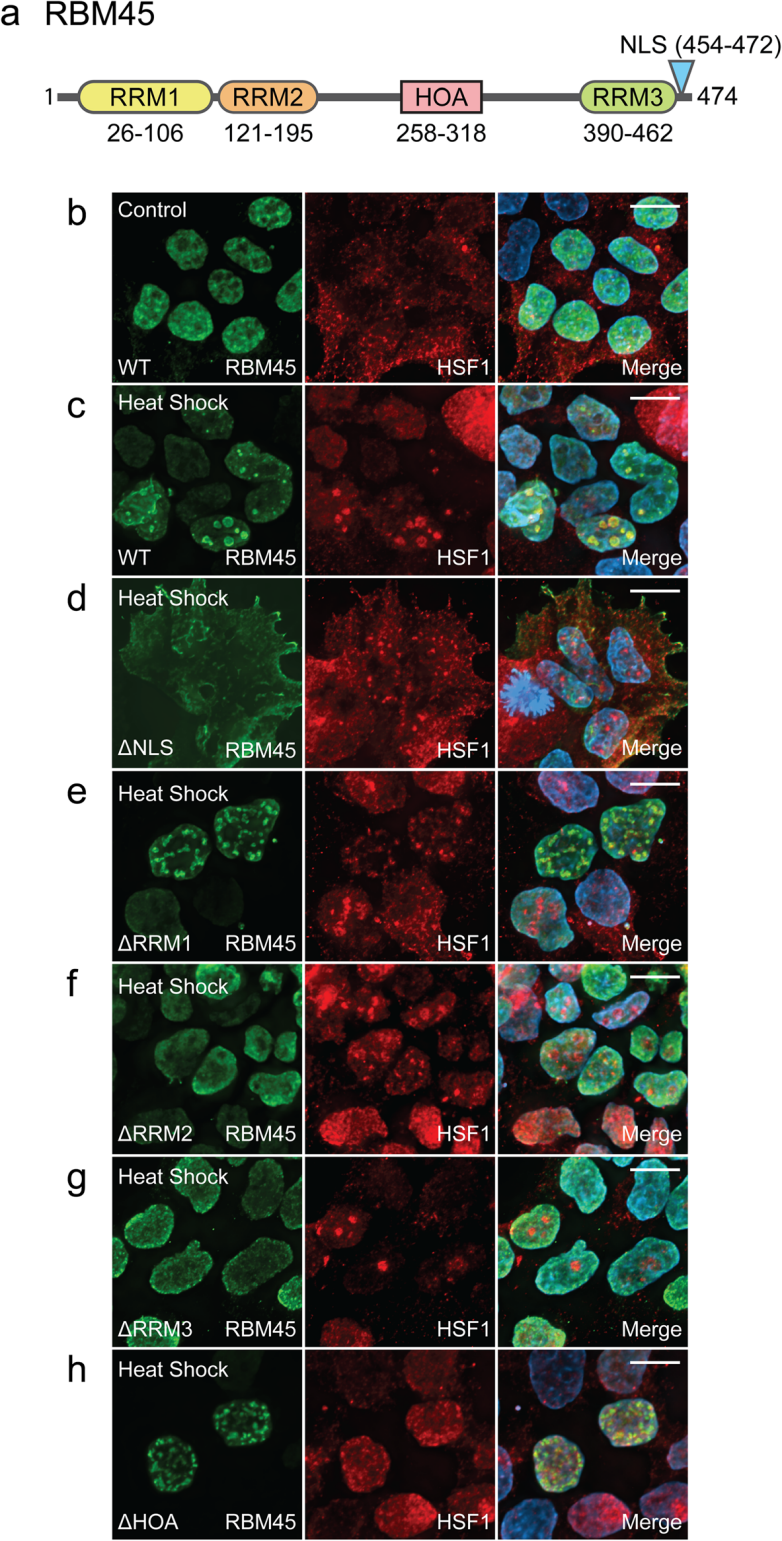
Domains required for RBM45 incorporation into nuclear stress bodies (NSBs). **a** Schematic showing functional domains and their position in the full-length RBM45 protein. RRM = RNA recognition motif, HOA = homo-oligomer assembly domain, NLS = nuclear localization sequence. HEK293 cells were transfected with constructs encoding N-terminally HA-tagged wild-type (WT) or domain-modified forms of RBM45 as indicated to determine which domains of the protein are necessary for incorporation into NSBs. **b** In unstressed cells, the distribution of WT RBM45 is diffuse and nuclear and no NSBs are observed. **c** Heat shock (42 °C for 2 h) leads to the robust formation of NSBs that are positive for wild-type (WT) RBM45 and the NSB marker HSF1. **d** Removal of the RBM45 NLS (∆NLS) leads to its sequestration in the cytoplasm and prevents its association with NSBs during heat shock. **e** Removal of RRM1 (∆RRM1) does not alter the association of RBM45 with NSBs. **f** Removal of RRM2 (∆RRM2) prevents the association of RBM45 with NSBs. **g** Removal of RRM3 (∆RRM3) prevents the association of RBM45 with NSBs. **h** Removal of the RBM45 HOA domain (∆HOA) does not alter the association of RBM45 with NSBs. For all images, scale bar = 5 μm

RBM45 is a 474 amino acid protein with 3 RNA recognition motifs (RRMs), a bipartite nuclear localization sequence (NLS), and a homo-oligomer assembly (HOA) domain that mediates its self-association/oligomerization and interaction with other proteins (Fig. 3a) [4, 6]. To identify RBM45 domains required for association with NSBs, we examined the co-localization of NSBs and HA-tagged full-length RBM45, several domain deletion mutant RBM45 constructs, and a nuclear localization sequence (NLS)-disrupting RBM45 mutant construct following heat shock. In unstressed cells, full-length HA-tagged RBM45 exhibited a diffuse nuclear localization and no HSF1-positive NSBs were observed (Fig. 3b). A similar result was observed under basal conditions for all RBM45 deletion constructs tested except the ΔNLS construct, which was retained in the cytoplasm (Fig. S6). Heat shock led to the incorporation of full-length, wild-type RBM45 in HSF1-positive NSBs (Fig. 3c). Disruption of the C-terminal bipartite NLS of RBM45 led to cytoplasmic sequestration of the protein and this was sufficient to abrogate the association of RBM45 with NSBs (Fig. 3d). Removal of the N-terminal RRM (RRM1) did not alter the association of RBM45 and NSBs (Fig. 3e), nor did removal of the protein’s HOA domain (Fig. 3h). In contrast, removal of either RRM2 or RRM3 was sufficient to prevent RBM45 from associating with NSBs (Fig. 3f and g). Our data, therefore, suggests a direct interaction of RBM45 with NSB SatIII transcripts via RRM2 and RRM3, rather than by indirect association with other NSB proteins.

### Chronic stress promotes RBM45 nuclear aggregation

**Fig. 4:**
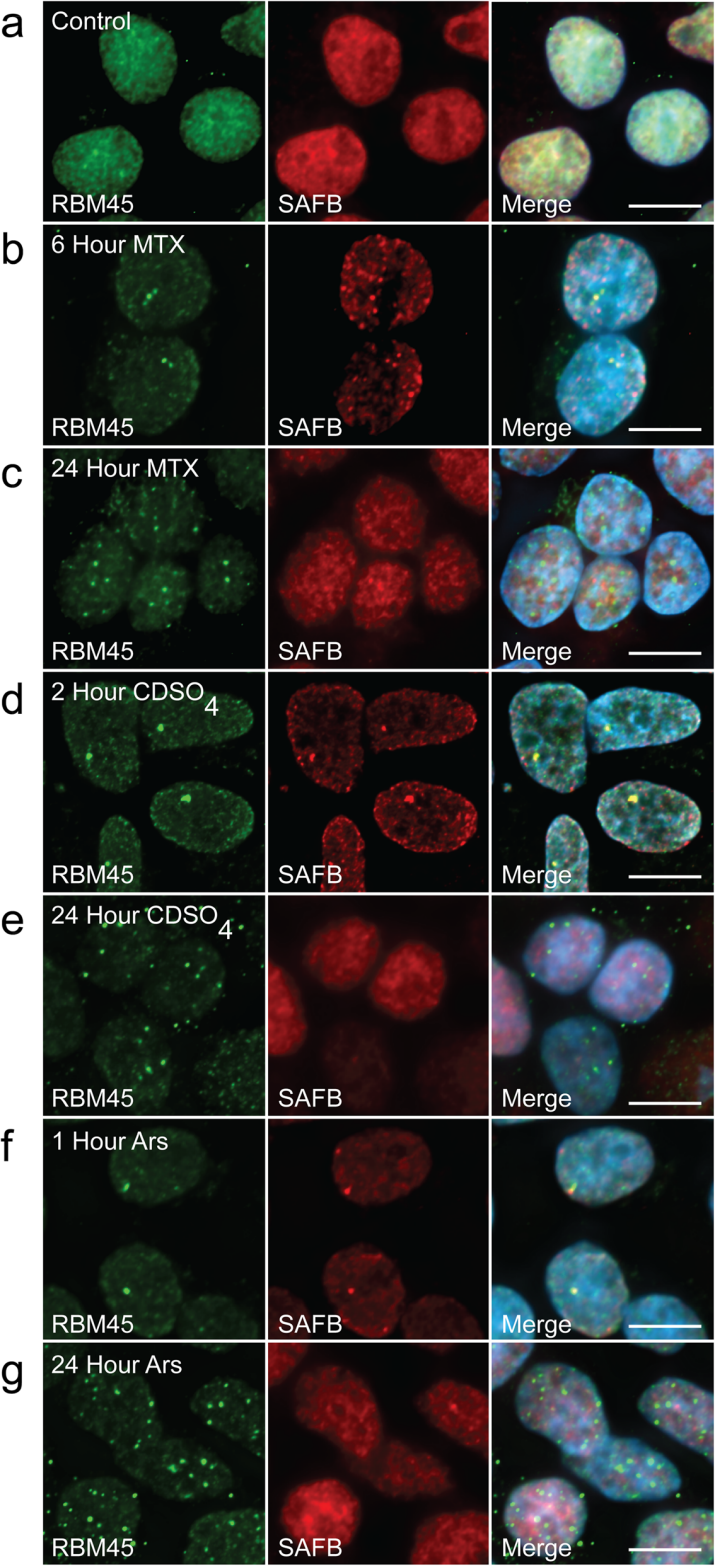
Chronic stress promotes RBM45 nuclear inclusion formation. HEK293 cells were treated with three separate cellular stressors for varying durations to examine the effects of chronic and acute cellular stress on the subcellular distribution of RBM45 and nuclear stress body (NSB) formation. **a** In untreated cells, the distribution of RBM45 is diffuse and nuclear and no SAFB-positive NSBs are visible. **b** Acute treatment (6 h) with the genotoxic stressor mitoxantrone (MTX; 5 μM) induces the formation of RBM45- and SAFB-positive NSBs. **c** Chronic treatment with MTX (24 h; 1 μM) leads to the formation of RBM45 inclusions via NSB formation. At 24 h, RBM45 nuclear inclusions are no longer positive for the NSB marker SAFB. (d and e) Similar results were obtained with acute (**d**) and chronic (**e**) treatment of cells with the oxidative stressor cadmium sulfate (CdSO_4_; 30 μM [acute] or 5 μM [chronic]) and the NSB marker SAFB. **f** and **g** Similar results were also obtained for acute and chronic treatment of HEK293 cells with sodium arsenite (Ars; 1 mM [acute; g] or 0.1 mM [chronic; h]). For all images, scale bar = 5 μm

We next asked whether persistent association with NSBs is sufficient to promote nuclear RBM45 inclusion formation. Nuclear RBM45 inclusions occur in ALS, FTLD, and AD [1, 2] and cellular stress has previously been shown to cause the formation of insoluble inclusions containing other ALS/FTLD-linked RBPs such as TDP-43 and FUS [20, 23, 24]. The formation of insoluble RBP inclusions may result from the persistent association of these proteins with cytoplasmic SGs. An analogous process occurring via RBM45 association with NSBs during chronic cellular stress could lead to the formation of RBM45 nuclear inclusions. To understand the effects of acute and chronic stress on the distribution and aggregation of RBM45, we used a variety of cellular stressors at differing concentrations and for varying durations. Following acute and chronic stressor treatments, we performed immunocytochemistry for endogenous RBM45 and NSBs. As in prior experiments, under basal conditions, RBM45 was diffusely localized throughout the nucleus and no NSBs were visible (Fig. 4a). Acute treatment with the genotoxic stressor MTX (5 μM for 6 h) led to the formation of RBM45-positive NSBs (Fig. 4b). To chronically stress cells with MTX, we tested a range of MTX concentrations (0.5–5 μM), seeking to find the maximum dose that did not adversely affect cellular viability. Using a dose of 1 μM MTX for 24 h, we found that chronic stress led to an increase in the size, staining intensity, and number of RBM45 foci in the nucleus of HEK293 cells, suggestive of the formation of RBM45 inclusions (Fig. 4c). These inclusions were no longer positive for NSB marker proteins. We used a similar approach to compare the effects of acute and chronic stress resulting from treatment with the cadmium sulfate (acute – 30 μM, 2 h; chronic – 5 μM, 24 h), and sodium arsenite (acute – 1 mM, 1 h; chronic – 0.1 mM, 24 h). We found that acute stress lead to the formation of SAFB/RBM45-positive NSBs (Fig. 4d, f). In contrast, chronic stress led to the formation of large, persistent RBM45-positive nuclear inclusions that were negative for NSB marker proteins (Fig. 4e, g).

NSB formation leads to the concentration of NSB proteins at sites containing SatIII transcripts, with attendant depletion of these proteins from their normal cellular milieu. Although acute cellular stress did not alter NSB protein levels (Fig. S2), we observed that the redistribution of proteins to NSBs led to a decrease in total nuclear NSB protein signal by quantitative immunocytochemistry (by changing from more diffuse to distinct puncta staining). We used this approach to quantify changes in RBM45 and SAFB immunoreactivity during conditions of acute and chronic stress. To visualize changes in RBM45 and SAFB on the same scale, we converted the values for each protein to Z scores. As expected, acute stress by MTX (5 μM for 6 h), CdSO_4_ (30 μM for 2 h), and sodium arsenite (1 mM for1 hour) all significantly reduced the total nuclear RBM45 signal compared to untreated cells (*p* < 1 × 10^− 11^ for MTX, *p* < 1 × 10^− 9^ for CDSO_4_, and *p <* 0.01 for sodium arsenite; Fig. S7A). These acute stressors likewise significantly reduced the total nuclear SAFB signal compared to untreated cells (*p* < 1 × 10^− 8^ for MTX, *p* < 1 × 10^− 9^ for CDSO_4_, and *p <* 1 × 10^− 12^ for sodium arsenite; Fig. S7B). By contrast, NSBs dissipated over the course of our chronic stress conditions (1 μM MTX, 5 μM CdSO_4_, or 0.1 mM sodium arsenite for 24 h) (Fig. 4c, e, g). We could detect this change as a significant increase in total nuclear SAFB signal for two of our three chronic stress conditions compared to the corresponding acute stressor (*p <* 0.01 for acute MTX versus chronic MTX and acute sodium arsenite, but *p* > 0.05 for acute versus chronic CdSO_4_; Fig. S7B), as well as a non-significant difference between untreated cells and these chronic stress conditions (*p* > 0.05 for untreated versus chronic MTX and sodium arsenite, but *p* < 1 × 10^− 4^ for untreated versus chronic CdSO_4_; Fig. S7B). Chronic stress led to the formation of nuclear RBM45 inclusions (Fig. 4c, e, g) and we could detect this change as a significant decrease in total nuclear RBM45 signal in cells in our chronic stress conditions compared to untreated cells (*p <* 1 × 10^− 9^ for untreated versus chronic MTX and *p* < 0.01 for untreated versus chronic CdSO_4_, and sodium arsenite; Fig. S7A). We also observed that the total nuclear RBM45 signal did not differ between individual acute versus chronic stress conditions, owing to RBM45’s confinement in NSBs and nuclear inclusions, respectively (*p* > 0.05 for acute versus chronic MTX, acute versus chronic CdSO_4_, and acute versus chronic sodium arsenite; Fig. S7A).

**Fig. 5:**
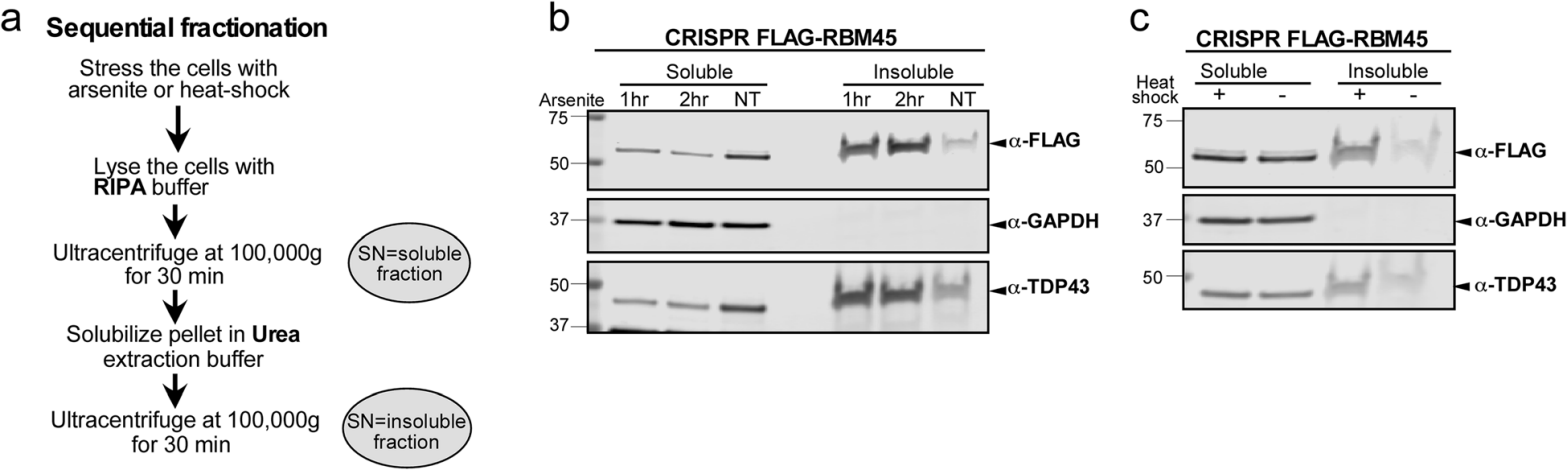
RBM45 solubility during conditions of cellular stress. **a** Workflow to obtain soluble and insoluble total protein extracts from untreated and stressed HEK293 cells expressing endogenous, N-terminally FLAG-tagged RBM45. “SN” = supernatant. **b** Western blotting for FLAG-tagged RBM45 (α-FLAG), GAPDH (negative insoluble fraction control), and TDP-43 (positive insoluble fraction control) following sodium arsenite treatment (1 mM for 1 or 2 h). **c** FLAG-RBM45, GAPDH and TDP-43 solubility following heat shock (1 h at 42 °C)

The RBM45 inclusions observed following chronic stress may reflect a change in RBM45 solubility. Because protein overexpression via transient transfection can lead to the formation of protein inclusions independent of cellular stress, we examined the solubility of endogenous RBM45 at physiological expression levels. For these experiments, we used CRISPR-Cas9 genome editing to append a 3x FLAG tag to the N-terminus of the endogenous RBM45 gene. We performed extensive characterization of CRISPR-Cas9-edited RBM45 HEK293 cells lines and found a non-significant reduction in RBM45 expression at the RNA level via qPCR in our CRISPR-RBM45 lines compared to unedited control cell lines (*p* > 0.05; Fig. S8A). We also observed no effects on nuclear morphology (Fig. S8B), no differences in RBM45 subcellular localization (Fig. S8B), or cellular morphology (Fig. S8B), suggesting that our CRISPR-cas9 genome editing and expression of HA-tagged RBM45 did not interfere with cellular physiology or RBM45 function. Soluble and insoluble protein extracts were obtained from FLAG-RBM45 cell lines as outlined in Fig. 5a. The purity of the insoluble extract was determined by blotting for GAPDH, which is not a component of the insoluble protein fraction in stressed or unstressed cells. We used TDP-43, which increases in the insoluble fraction following cellular stress, as a positive control [43]. As shown in Fig. 5b, heat shock or treatment with sodium arsenite led to a significant increase (*p* < 0.01 for both comparisons) in the amount of insoluble RBM45 (denoted using anti-FLAG tag antibody). For treatment with sodium arsenite, we observed that increasing the stress duration led to increased levels of insoluble RBM45 (*p* < 0.05). Treatment with sodium arsenite led to a concomitant decrease in the amount of soluble RBM45 protein (*p* < 0.01), while heat shock did not alter the levels of soluble RBM45 (*p* > 0.05). A similar pattern was observed for TDP-43, where sodium arsenite stress significantly increased the amount of insoluble TDP-43 compared to unstressed cells (*p* < 0.01 for both comparisons), while heat shock increased the amount of insoluble TDP-43 (*p <* 0.01), but did not alter the amount of soluble TDP-43 (*p* > 0.05).

**Fig. 6:**
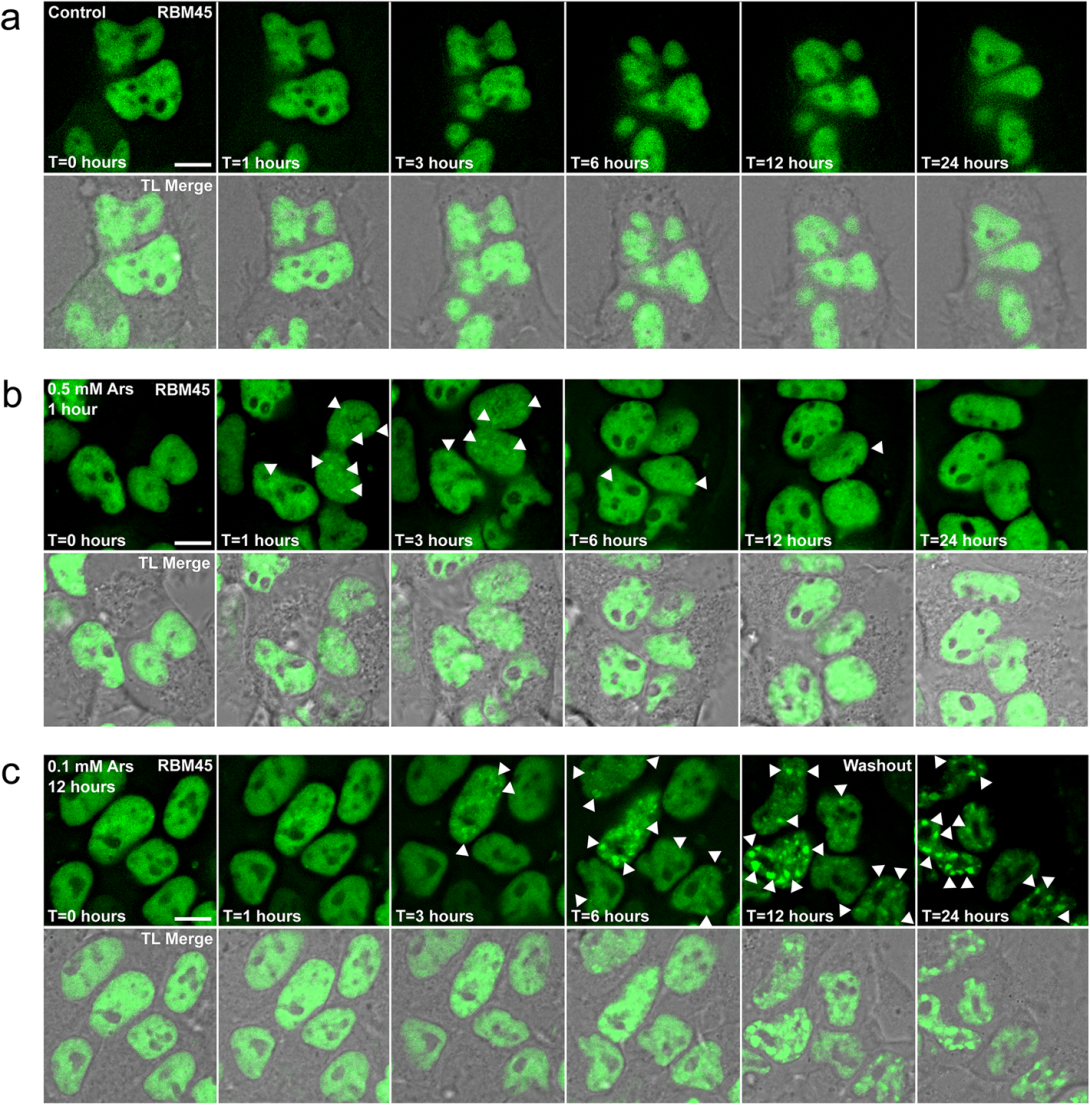
Live-cell imaging of RBM45 nuclear stress body (NSB) and nuclear inclusion formation during conditions of cellular stress. HEK293 cells induced to express N-terminal GFP-tagged RBM45 were used to visualize the subcellular distribution of RBM45 during normal conditions and during acute and chronic cellular stress. **a** In untreated cells, RBM45 is predominantly nuclear and diffusely localized throughout the nucleus. **b** Treatment with 0.5 mM sodium arsenite for 1 h causes the formation of numerous RBM45-positive nuclear stress bodies (NSBs; arrowheads). Cells were treated with 0.5 mM sodium arsenite at t = 0 h. Under conditions of acute cellular stress, NSB formation is reversible, most RBM45-positive NSBs have dissipated by t = 6 h, and RBM45 remains diffusely localized throughout the nucleus for the remainder of the experiment. **c** Chronic cellular stress leads to the entrapment of RBM45 in nuclear inclusions. Cells were treated with 0.1 mM sodium arsenite for 12 h to assess the effect of chronic cellular stress on RBM45 subcellular distribution. Because of the lower concentration of sodium arsenite, the time course of NSB formation is altered. Following removal of sodium arsenite (panel “Washout”), RBM45 remains in nuclear inclusions for the remaining 12 h duration of the experiment (arrowheads). For all panels, scale bar = 5 μm

To understand the temporal dynamics of RBM45 NSB association and inclusion formation, we used live-cell imaging of HEK293 cells expressing N-terminally GFP-tagged RBM45 under the control of the Tet repressor. Addition of 10 μg/ml doxycycline to the culture medium led to robust expression of EGFP-RBM45. We used live-cell imaging to measure the dynamics of RBM45 during cellular stress. In untreated control cells, RBM45 maintained its diffuse nuclear localization for the 24 h duration of the live-cell imaging experiments (Fig. 6a). No RBM45 nuclear inclusions were observed during this period. In contrast, acute treatment with 0.5 mM sodium arsenite led to the formation of RBM45-positive NSBs (Fig. 6b). These foci emerged within 1 h of treatment (arrowheads in Fig. 6b) and were almost completely eliminated 5 h after removal of the stressor (Fig. 6b, T = 6 h panel). At the completion of the experiment, no RBM45-positive NSBs were visible (Fig. 6b, T = 24 h panel). In contrast, chronic treatment with 0.1 mM sodium arsenite for 12 h led to the formation of large nuclear RBM45 inclusions that persisted for the 24-h duration of the experiment (Fig. 6c, T = 12 h and T = 24 h panels). These inclusions were larger, more numerous, and brighter than those observed during acute stress conditions (compare Fig. 6c T = 12 h panel to Fig. 6b T = 3 h panel). The size and number of the RBM45 inclusions increased over time in chronic stress, unlike the acute stress condition (Fig. 6c T = 6, 12, and 24 h panels). The presence of multiple, large RBM45 inclusions also impacted nuclear morphology (Fig. 6c panel T = 12 and T = 24 h).

### Quantification of RBM45 NSB pathology in ALS, FTLD, and AD subjects

**Fig. 7:**
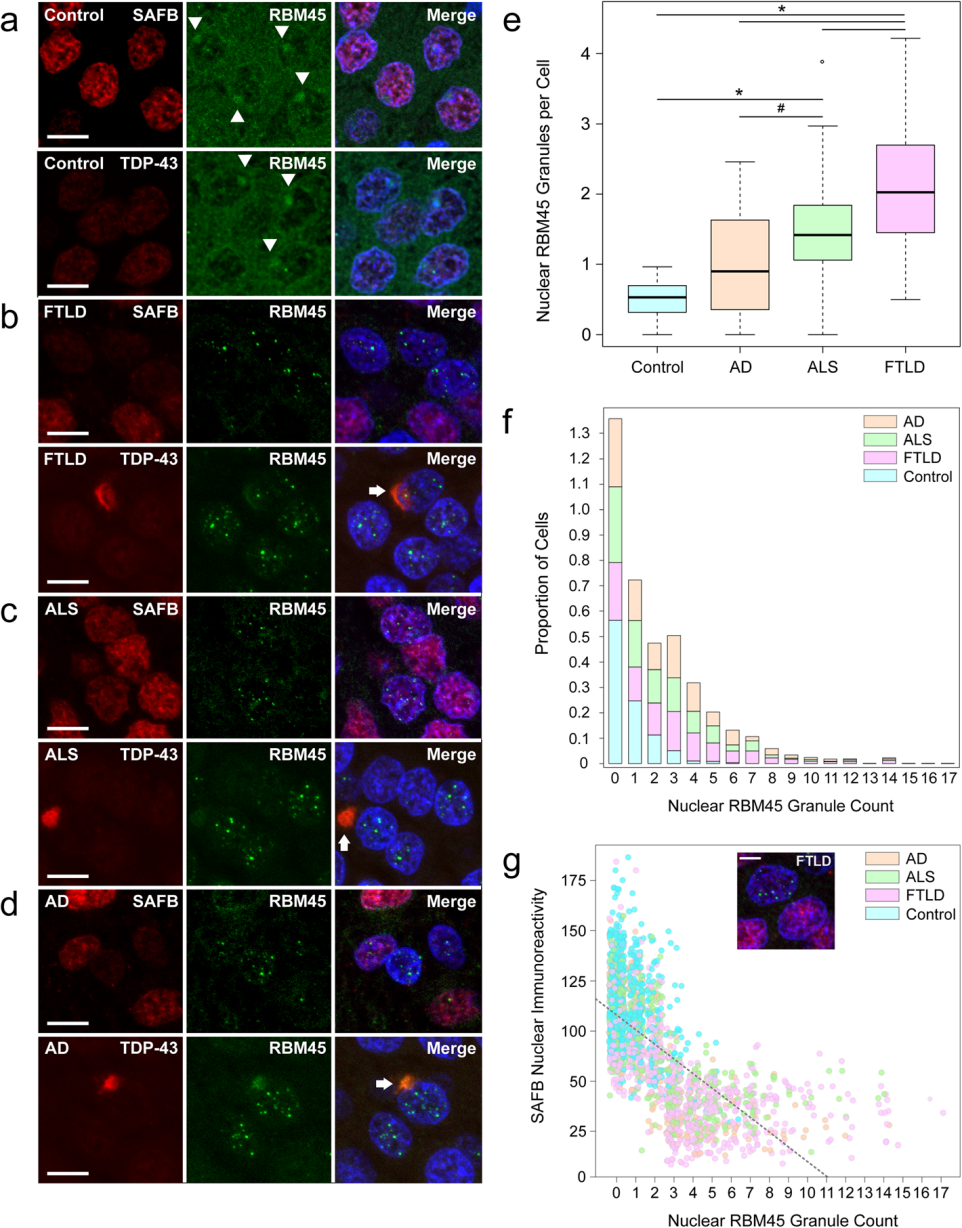
RBM45 subcellular distribution and inclusion pathology in FTLD-TDP, ALS, and AD. Immunohistochemistry for RBM45, SAFB, and TDP-43 was performed in hippocampal dentate gyrus from non-neurologic disease control, FTLD-TDP, ALS, and AD subjects. **a** Control subjects showed strong immunoreactivity for SAFB, TDP-43, and RBM45, with RBM45 immunoreactivity concentrated in the perinucleolar region (arrowheads) and SAFB and TDP-43 diffusely localized throughout the nucleus. **b** RBM45 immunoreactivity was predominantly in nuclear inclusions in FTLD dentate gryus cells, with attendant loss of cytoplasmic and perinucleolar immunoreactivity. Nuclear RBM45 inclusions lack SAFB and TDP-43. Dentate gyrus cells containing RBM45 nuclear inclusions also exhibit reduced SAFB immunoreactivity. Several TDP-43 and RBM45-positive cytoplasmic inclusions were found in FTLD subjects (arrow). **c** As in (**b**), but for ALS subjects. Nuclear RBM45 inclusions lack SAFB or TDP-43, and cytoplasmic inclusions contain RBM45 and TDP-43 (arrow). **d** As in (**b**), but for AD subjects. Nuclear RBM45 inclusions lack SAFB and TDP-43, and cytoplasmic inclusions contain RBM45 and TDP-43 (arrow). **e** Boxplot showing the number of RBM45 nuclear inclusions by group (# = *p* < 0.01, * = *p* < 1 × 10^− 6^). **f** Stacked bar plot showing the number of RBM45 nuclear inclusions per cell by subject group. Most dentate gyrus cells from control subjects have no inclusions, while FTLD-TDP, ALS, and AD subjects have significantly more granules per cell across the range of observed values. **g** The relationship between RBM45 nuclear inclusions and SAFB immunoreactivity is shown, along with the regression line. Regardless of disease state, with increasing numbers of RBM45 nuclear inclusions, SAFB immunoreactivity decreases, particularly at ≥3 granules per cell. The relationship between these two variables is statistically significant (*p* < 1 × 10^− 16^). In the scatterplot shown, jitter is added to the points to more clearly visualize the data, but the separation between inclusion integer numbers is still seen. The inset image shows an example of adjacent cells in an FTLD-TDP subject where one cell with no RBM45 granules has strong SAFB nuclear immunoreactivity, while the neighboring cell has several nuclear RBM45 inclusions and low SAFB nuclear immunoreactivity. In (**a-d**), the scale bar = 20 μm, while in the inset in (**g**) the scale bar = 5. For all panels, FTLD = FTLD-TDP

To characterize cell-type specific patterns of RBM45 pathology in ALS, FTLD-TDP, and AD, we quantified RBM45 nuclear and cytoplasmic pathology across these diseases and measured the co-localization of the NSB marker protein SAFB with RBM45 nuclear inclusions. We first performed immunohistochemistry for RBM45, SAFB, and TDP-43 in the dentate gyrus of non-neurological disease controls, FTLD-TDP, ALS, and AD (Fig. 7a-d). We focused our efforts on the dentate gyrus as RBM45 nuclear inclusions are significantly more prevalent in the dentate gyrus than in hippocampal pyramidal neurons [1]. Subject demographics are shown in Table 1. We used an image analysis pipeline to extract the number of cells, the number of RBM45 nuclear inclusions, and SAFB immunoreactivity by confocal microscopy (see Methods). Cytoplasmic RBM45 inclusions were counted manually. We counted a total of 8 fields per subject, corresponding to a total of 10,640 cells across all groups. In non-neurologic disease controls, RBM45 exhibited diffuse nuclear and strong perinucleolar immunoreactivity in dentate gyrus granule cells (Fig. 7a, arrowheads). These cells also exhibited strong, diffuse nuclear SAFB immunoreactivity. In contrast, granule cells in FTLD-TDP, ALS, and AD patients typically exhibited multiple RBM45 nuclear inclusions with a corresponding loss of nuclear/perinucleolar RBM45 immunoreactivity (Fig. 7b-d, respectively). RBM45 nuclear inclusions were negative for SAFB, consistent with our in vitro results in chronic stress conditions. Cytoplasmic RBM45 inclusions were occasionally observed in dentate gyrus granule cells and were positive for TDP-43 (Fig. 7b-d, bottom panels [arrows]), but negative for SAFB.

We quantified the number of nuclear and cytoplasmic RBM45 inclusions in dentate gyrus granule cells of each subject. The distribution of nuclear inclusions exhibited a strong rightward skew, with many cells containing zero inclusions. Accordingly, we used the non-parametric Mann-Whitney U test to assess the differences in RBM45 inclusion counts between groups. FTLD-TDP subjects exhibited significantly more RBM45 inclusions than non-neurologic disease controls, AD, or ALS subjects (Fig. 7e; *p* < 1 × 10^− 6^ for control and AD and *p* < 0.01 for ALS versus FTLD-TDP, respectively). ALS subjects had significantly more nuclear inclusions than control or AD subjects (*p* < 1 × 10^− 6^ and *p* < 0.01, respectively). Because non-neurologic disease controls had significantly more hippocampal dentate gyrus cells than FTLD-TDP, ALS, or AD subjects, we repeated this comparison after normalizing RBM45 inclusions to the number of cells and found that FTLD-TDP had significantly more nuclear inclusions per cell than ALS, AD, and control subjects (*p* < 1 × 10^− 6^, *p* < 1 × 10^− 8^, respectively). ALS subjects had significantly more RBM45 nuclear inclusions than control and AD subjects (*p* < 1 × 10^− 6^ and *p* < 0.01, respectively). The comparison between AD and non-neurologic disease controls did not reach statistical significance following multiple testing correction (*p* = 0.015). We observed that cells containing RBM45 nuclear inclusions often had multiple inclusions (in some instances more than 10 per nucleus), irrespective of disease state (Fig. 7f). AD and ALS subjects showed comparable proportions and numbers of inclusions per cell, while the vast majority of cells from control subjects (> 85%) show 1 or fewer nuclear inclusions. The proportion of hippocampal cells containing at least 1 nuclear RBM45 inclusion was significantly greater than the proportion of cells containing at least 1 cytoplasmic inclusion (*p* < 1 × 10^− 8^). Cytoplasmic RBM45 inclusions were not observed in non-neurologic disease controls. Summary statistics for RBM45 nuclear and cytoplasmic pathology in the dentate gyrus are shown in Table 2.

**Table 2:** Summary statistics from tissue immunohistochemistry image analysis. The table shows the number of individuals and cells counted for each tissue region and subject group. The number of neurons or glial cells and RBM45 nuclear and cytoplasmic inclusions were counted as described in the Methods section. For each subject, a total of 8 fields per region (hippocampal dentate gyrus or lumbar spinal cord) were evaluated. FTLD-TDP = frontotemporal lobar degeneration with TDP-43 proteinopathy

Group	N	Total Cells	Mean Cells/ Field	SEM Cells/ Field	Mean Nuclear RBM45 Inclusions/Cell	SEM Nuclear RBM45 Inclusions/Cell	Mean Cytoplasmic RBM45 Inclusions/Cell	SEM Cytoplasmic RBM45 Inclusions/Cell
**Dentate Gyrus Granule Cells**								
Control	10	4145	51.81	4.92	0.44	0.09	0	0
AD	5	1590	39.75	8.32	0.98	0.34	0.044	0.051
ALS	8	2943	45.98	5.98	1.49	0.27	0.048	0.055
FTLD-TDP	6	1962	40.92	5.81	2.38	0.23	0.109	0.072
**Spinal Cord Glial Cells**								
Control	9	693	9.62	1.18	0.23	0.06	0	0
ALS	15	2689	22.35	1.74	1.04	0.18	0.11	0.47
**Spinal Cord Motor Neurons**								
Control	9	308	4.29	0.53	0	0	0	0
ALS	15	351	2.91	0.42	0	0	0.15	0.14

In subjects with nuclear RBM45 inclusions, a bimodal pattern of SAFB immunoreactivity was seen wherein cells with multiple RBM45 nuclear inclusions had low SAFB immunoreactivity and cells without RBM45 nuclear inclusions had higher SAFB immunoreactivity. We quantified this relationship (Fig. 7g) and used linear regression to assess its significance. Across all groups, increasing numbers of RBM45 nuclear inclusions per cell were associated with decreasing SAFB immunoreactivity (*p <* 1 × 10^− 16^). SAFB nuclear immunoreactivity was lowest when cells had ≥3 RBM45 nuclear inclusions (Fig. 7g, inset).

**Fig. 8:**
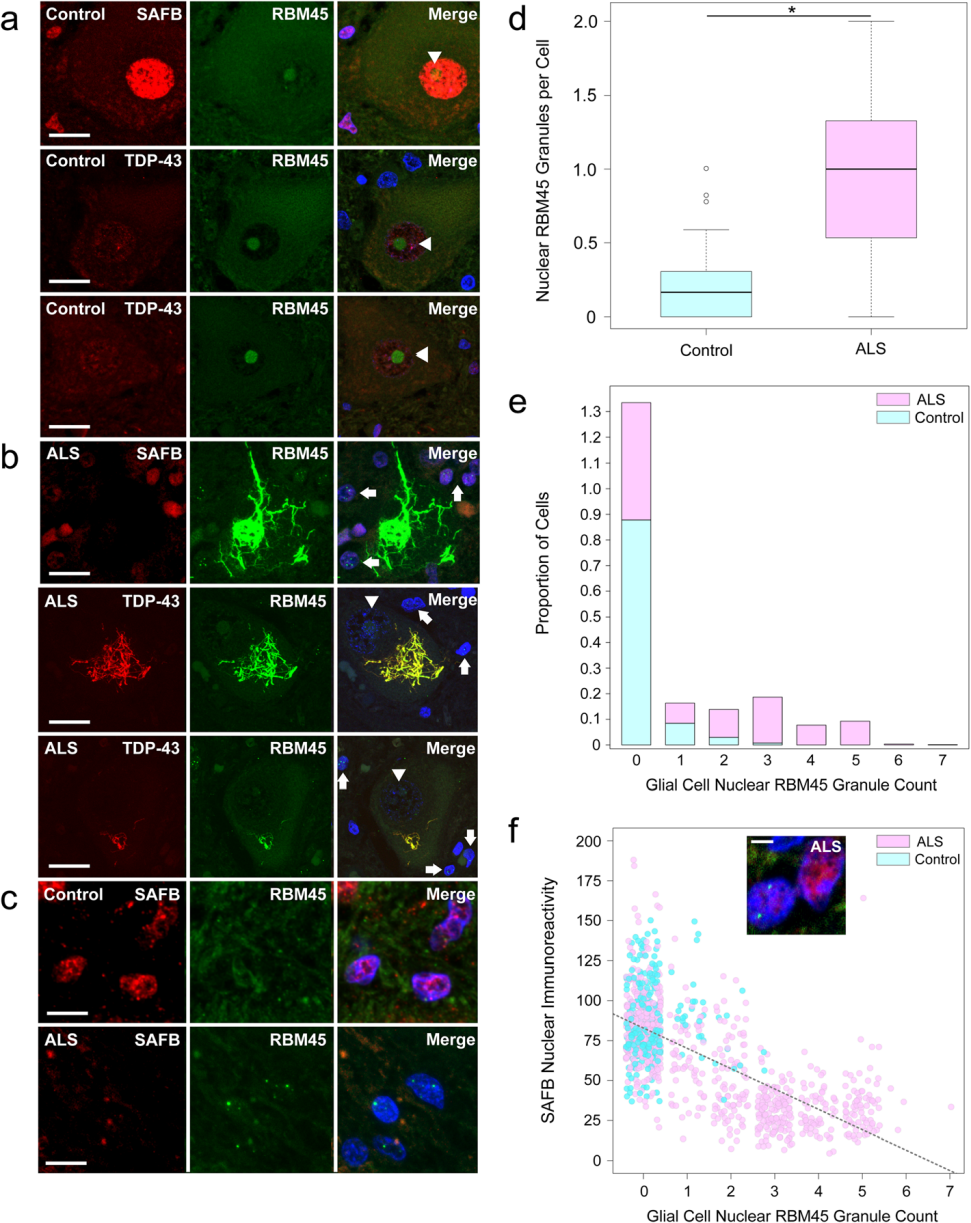
RBM45 subcellular distribution and inclusion pathology in lumbar spinal cord. Immunohistochemistry for RBM45 with either SAFB or TDP-43 was performed in lumbar spinal cord sections from ALS and non-neurologic disease control subjects. **a** RBM45, SAFB, and TDP-43 immunoreactivity were measured in motor neurons and glial cells of non-neurologic disease control subjects. RBM45 exhibited strong nucleolar immunoreactivity in motor neurons (arrowheads), while SAFB and TDP-43 immunoreactivity were diffusely nuclear. **b** In ALS patients, motor neuron nucleolar RBM45 immunoreactivity was reduced (arrowheads) and large, skein-like cytoplasmic inclusions were observed. These inclusions were positive for TDP-43 but negative for SAFB. ALS patient glial cells frequently contained multiple RBM45 nuclear inclusions (arrows). **c** Glial cells from non-neurologic disease control (top) and ALS (bottom) subjects. Glial cells from ALS subjects frequently contained 1 or more nuclear RBM45 inclusions. The presence of glial RBM45 nuclear inclusions was associated with reduced SAFB immunoreactivity. **d** Boxplot showing the number of glial nuclear RBM45 inclusions in control and ALS subjects. For the comparison shown, * = *p* < 1 × 10^− ^^8^. **e** Stacked bar plot showing the proportion of cells (Y axis) with the given number glial nuclear RBM45 inclusions (X axis) across groups. ALS subjects have significantly more inclusions per glial cell than control subjects, who rarely exhibit RBM45 glial cell pathology. **f** The relationship between glial RBM45 nuclear inclusions and SAFB immunoreactivity is shown, along with the regression line. Increasing numbers of RBM45 nuclear inclusions are associated with reduced SAFB immunoreactivity, particularly at ≥3 granules per cell. The relationship between these two variables is statistically significant (*p* < 1 × 10^−6^). In the scatterplot shown, jitter is added to the points to more clearly visualize the data, but the separation between inclusion integer numbers can still be seen. The inset image shows an example of adjacent glial cells in an ALS patient where one cell without RBM45 nuclear inclusions has strong SAFB nuclear immunoreactivity, while the neighboring cell with RBM45 nuclear inclusions shows reduced SAFB immunoreactivity. In (**a** and **b**) the scale bar = 20 μm, in (**c**) the scale bar = 5 μm, and for the inset in (**f**) the scale bar = 2.5 μm

We applied the same analysis to lumbar spinal cord tissue sections from ALS and non-neurologic disease controls. In motor neurons from non-neurologic disease control subjects, RBM45 immunoreactivity was largely nucleolar (Fig. 8a, arrowheads) and SAFB and TDP-43 immunoreactivity was diffuse. RBM45, TDP-43, or SAFB nuclear and cytoplasmic inclusion pathology was absent in control subjects (Fig. 8a). In contrast, ALS patients frequently exhibited skein-like cytoplasmic RBM45 inclusion pathology that co-localized with TDP-43 but not SAFB (Fig. 8b). We noted a decrease in RBM45 nucleolar immunoreactivity in spinal cord motor neurons containing cytoplasmic RBM45 inclusions (Fig. 8b, arrowheads). Despite altered RBM45 motor neuron nucleolar immunoreactivity, we did not detect nuclear RBM45 inclusions in motor neurons of ALS subjects (Fig. 8b, Table 2). Instead, we frequently observed RBM45 nuclear inclusions in adjacent glial cells in ALS, but not in non-neurologic disease controls (Fig. 8b and c [arrows]; Table 2).

We quantified lumbar spinal cord glial cells and glial nuclear RBM45 inclusions in individual image fields. A total of 3382 lumbar spinal cord glial cells across 26 subjects were counted using this approach. Glial cells in ALS patients contained significantly more RBM45 nuclear inclusions per cell than those from non-neurologic disease controls (Fig. 8d, *p <* 1 × 10^− 8^). While approximately 90% of glial cells in control spinal cord exhibited no RBM45 nuclear inclusions, greater than 50% of ALS glial cells had one or more RBM45 nuclear inclusions (Fig. 8e). The proportion of spinal cord glial cells containing nuclear RBM45 inclusions was significantly greater than the proportion of spinal cord glial cells with cytoplasmic RBM45 inclusions (*p* < 1 × 10^− 4^; Table 2). Similar to the hippocampus, glial cells that contained multiple RBM45 nuclear inclusions displayed reduced SAFB immunoreactivity (Fig. 8f, inset). We explored the relationship between nuclear RBM45 inclusion number and SAFB immunoreactivity and found that greater numbers of RBM45 inclusions per cell were associated with decreasing SAFB nuclear immunoreactivity (Fig. 8f, *p* < 1 × 10^− 6^). SAFB nuclear immunoreactivity was lowest when cells had ≥3 RBM45 nuclear inclusions (Fig. 8f, inset). Summary statistics for the image analysis of human spinal cord tissue are shown in Table 2.

## Discussion

The goals of this study were to further characterize the normal functions of RBM45, define the mechanisms by which RBM45 forms nuclear inclusions, and quantify cell type-specific patterns of RBM45 inclusion pathology in FTLD-TDP, ALS, and AD. We found that RBM45 associates with nuclear stress bodies (NSBs), stress-induced protein-RNA complexes, in response to a diverse array of cellular stressors as part of its normal functions. This association is mediated by the protein’s nuclear localization sequence and RNA recognition motifs (RRM) 2 and 3. In addition, the chronic entrapment of RBM45 in NSBs was sufficient to promote nuclear RBM45 inclusion formation, even when other NSB proteins had disassociated from these complexes. In human CNS tissue, nuclear RBM45 inclusions were frequently found in ALS, FTLD-TDP, and AD in distinct cell types and this pathology occurs more frequently than cytoplasmic RBM45 inclusions. Nuclear RBM45 inclusions in post-mortem tissue lack NSB marker proteins, consistent with our in vitro models of chronic stress.

Aggregation and assembly into membraneless organelles is essential to the normal functions of many RBPs, and aids in regulating transcription, mRNA splicing, transport, and decay [58]. The assembly of RBPs, nucleic acids, and other factors into membraneless organelles acts to compartmentalize these components, leading to a high local concentration of enzymes and substrates of the associated biochemical reactions [58–60]. Our prior work demonstrated that RBM45 regulates mRNA processing and forms oligomeric complexes and interacts with other RBPs via an intrinsically disordered region termed the homo-oligomer assembly (HOA) domain [3, 4]. We, therefore, sought to determine whether RBM45 associates with an RBP-containing nuclear organelle. To this end, we examined the co-localization of RBM45 and several membraneless, RBP-containing organelles, including nuclear speckles, Cajal bodies, nuclear gems, and NSBs. Under basal conditions RBM45 does not co-localize with any of these organelles and, instead, exhibits a diffuse nuclear localization (Fig. 1). Subsequently, we observed that RBM45 coalesces into nuclear puncta following the onset of cellular stress and these puncta correspond to NSBs (Figs. 1 and 2).

NSBs are protein-RNA complexes that form in response to stress-induced transcription of satellite III (SatIII) repeats from pericentromeric heterochromatin [49]. The resultant SatIII transcripts act as scaffolds that recruit various RBPs to NSBs, notably the transcription factor HSF1 and the hnRNP SAFB, resulting in the appearance of several nuclear granules that disassemble following stressor removal [40, 49]. Despite a well-characterized mechanism of formation, the functions of NSBs have remained enigmatic. Current theory suggests that NSBs act as one component of a larger gene expression regulatory program in the cellular response to stress [49]. By sequestering various DNA/RNA binding proteins, such as RBM45, SAFB, and HSF1, NSBs are thought to alter gene expression through effects on both transcription and splicing. Aside from this general model, however, little is known about how NSBs alter the function of their constituent proteins during stress. NSBs are specific to primate cells and are not found in other organisms, including rodents [40, 49]. Consequently, the role of NSB proteins in the cellular response to stress may exhibit species-specific differences. Further studies are needed to understand the role of NSBs in the cellular response to stress and how incorporation into NSBs alters the gene regulatory functions of RBM45 and other NSB proteins.

RBPs can be recruited to NSBs by direct binding to SatIII transcripts or indirectly via protein-protein interactions with resident NSB proteins [49]. Our data indicates that recruitment of RBM45 to NSBs requires RNA binding motifs (RRM2 or RRM3) and does not require the HOA domain that mediates protein-protein interactions with other RBPs (Fig. 3). This suggests that RBM45 associates with NSBs via direct RNA binding. Prior studies demonstrated that RBM45 has a binding preference for poly (C) and poly (G) RNA [6]. The SatIII transcripts that act as the scaffolds for NSBs are themselves G- and C-rich [40, 41], consistent with our hypothesis that RBM45 incorporation into NSBs is a consequence of direct binding to SatIII transcripts. We observed that RBM45 is not required for the formation of NSBs (Fig. S5). This result is not unexpected given that the mechanism of NSB formation is transcription of SatIII DNA by HSF1 binding rapidly following stress onset [40, 49]. NSB formation also exhibits cell- and stressor-specific variability and even HSF1 itself is dispensable for NSB formation during certain types of cellular stress [32]. Thus, the primary function of RBM45 in NSBs appears to be removing the protein from its normal, diffuse nuclear localization, similar to other NSB proteins [49].

Previous studies reported that RBM45 associates with cytoplasmic stress granules and interacts with stress granule proteins, notably TDP-43 and FUS [4, 5]. We examined the distribution of RBM45 during conditions of cellular stress and found no evidence of RBM45 cytoplasmic translocation or association with cytoplasmic SGs (Fig. 1d, e and Fig. S1). Several factors may account for the conflicting claims regarding RBM45 and SGs made here and elsewhere. First, previous reports of RBM45 association with SGs were made using plasmid-based overexpression of RBM45 with a mutated, non-functional NLS [4, 5]. While such approaches establish that RBM45 can associate with SGs, they rely on artificially disrupting the normal subcellular localization of RBM45 and expressing the protein at non-physiological levels. Our study using wild-type RBM45 expression at physiologic levels failed to identify RBM45 association with cytoplasmic SGs under a diverse array of stress conditions (Fig. S1). Consistent with this observation, recent studies to comprehensively characterize the SG proteome failed to detect RBM45 within SGs [55, 61].

After identifying RBM45 as a component of NSBs, we asked whether chronic entrapment of the protein in these structures could seed nuclear RBM45 inclusion formation. RBM45 self-assembles into higher-order oligomers [4] and this behavior is likely essential to one or more of the protein’s normal functions, similar to other aggregation-prone RBPs [55, 62]. While acute stress results in transient RBM45 association with NSBs, chronic stress was sufficient to convert RBM45 into an insoluble form and irreversibly trap the protein in nuclear inclusions (Figs. 4, 5 and 6). Importantly, in the absence of SatIII transcripts, this phenomenon was not observed (Fig. S5), indicating that RBM45 nuclear aggregation and inclusion formation requires the initial formation of RBM45 containing NSBs. We also observed that TDP-43 and FUS are not recruited to NSBs (Fig. S3). This result suggests these proteins are not components of the nuclear RBM45 inclusions we observe in FTLD-TDP, ALS, and AD because they are not recruited to NSBs.

RBM45 nuclear inclusions that result from chronic stress lack NSB marker proteins, despite initial co-localization in NSBs (Fig. 4). Similarly, we did not observe co-localization of RBM45 and NSB marker proteins in ALS, FTLD, or AD tissue (Figs. 7 and 8). Several factors may account for these observations. The HOA domain that mediates RBM45 self-association is an intrinsically disordered peptide segment that promotes the assembly of RBM45 oligomers [4]. Chronic stress likely facilitates this process via compartmentalization in NSBs in a manner analogous to the sequestration and aggregation of TDP-43 and FUS in cytoplasmic SGs. HSF1 and SAFB do not oligomerize into large, macromolecular assemblies as part of their normal functions. HSF1 functions as a DNA binding-competent trimer and is only detectable in NSBs over a limited time course, specifically the early, but not late, phase of NSB lifetime [50]. SAFB likewise functions as a monomer or homodimer, but has not previously been shown to assemble into oligomeric complexes. It too, is detected over a limited time frame in the lifespan of a NSB, appearing approximately 1 h after HSF1 granules are detectable [50]. Despite the fact that RBM45, HSF1, and SAFB are all incorporated into NSBs, no direct physical interaction between these proteins has previously been observed [3]. The physical and temporal separation of these proteins, together with the low aggregation potential of HSF1 and SAFB, provide plausible explanations for the absence of these proteins in RBM45 nuclear inclusions resulting from chronic stress. RBM45 nuclear inclusions may nonetheless contain additional proteins and future studies will characterize the proteome of these structures.

A consistent finding from our in vitro and human tissue data is that nuclear RBM45 inclusions abrogate the normally diffuse localization of RBM45 protein in the nucleus (Figs. 4, 7, 8, S7). These results raise the possibility that RBM45 inclusions lead to loss of the normal functions of RBM45 by sequestering the protein from its normal sites of activity. RBM45 inclusions could, thus, contribute to cell death in disease via a “two-hit” model, wherein inclusions cause both toxic gain and loss of function. Such a model is well-established for other aggregation-prone RBPs in disease, including TDP-43 and FUS [21, 63, 64]. RBM45 regulates mRNA splicing and processing [3, 65] and perturbing these functions may, therefore, have broad effects on gene expression and cellular physiology. Studies of RBM45’s role in several biological processes support this prediction, showing that RBM45 regulates mitochondrial function [5], lipid metabolism [65], and the immune response [66]. However, further studies into the RNA targets of RBM45 are needed to more precisely predict how loss of the protein’s normal functions influences gene expression and other cellular and organismal phenotypes.

RBM45 is frequently observed in cytoplasmic and nuclear inclusions in neurons and glia in FTLD-TDP, ALS, and AD, but not in non-neurologic disease controls [1, 2]. To better understand cell type- and disease-specific patterns of RBM45 inclusion pathology in neurodegenerative diseases, we performed an extensive quantification of RBM45 pathology in ALS, FTLD-TDP, AD, and non-neurologic disease controls. In the hippocampal dentate gyrus, numerous RBM45 nuclear inclusions with a punctate morphology were observed in FTLD, AD, and ALS subjects, but not in non-neurologic disease controls. When we quantified the number of cells and RBM45 nuclear inclusions in each cell, we found that FTLD subjects had the highest proportion of cells with at least 1 nuclear inclusion and the highest average number of nuclear inclusions per cell. RBM45 nuclear inclusions were negative for TDP-43 or SAFB. Instead, we found that the presence of RBM45 nuclear inclusions correlated with disruption of the normal distribution and immunoreactivity of SAFB in ALS, FTLD, and AD (Figs. 7 and 8), similar to our results with chronic CdSO_4_ treatment (Fig. S7). Critically, this was not due to a general decrease in SAFB immunoreactivity, as neighboring cells without RBM45 nuclear inclusions showed strong SAFB immunoreactivity comparable to non-neurologic disease controls (Figs. 7g and Figs. 8f, inset). The distribution of RBM45 nuclear inclusions in dentate gyrus granule cells ranged from 0 to 17, with the majority of cells harboring > 5 inclusions found in FTLD subjects. This range of values shows good agreement with previous efforts to quantify the number of NSBs that form in response to different stressors in cultured cells. One study found that individual HeLa cells typically produced 1–20 NSBs in response to a variety of stressors, with 50% of cells harboring 1–5 NSBs [32]. We hypothesize that RBM45 nuclear inclusions in human post-mortem tissues denote the prior generation of NSBs in these cells with subsequent chronic stress. We observed RBM45 nuclear inclusions in some aged non-neurologic disease controls, though this may be due to the normal aging process as seen with other RBPs [67, 68].

We also quantified RBM45 pathology in the lumbar spinal cord of ALS and non-neurologic disease controls. As in our prior study, we found TDP-43-positive RBM45 cytoplasmic inclusions in motor neurons of ALS but not non-neurologic control subjects (Fig. 8). A key finding from this study is RBM45 nuclear inclusions show cell-type specificity in ALS spinal cord. Specifically, we observed RBM45 nuclear inclusions in ALS glial cells, but not motor neurons. RBM45 exhibits a predominantly nucleolar immunoreactivity in motor neurons in human CNS tissue that was not seen in any other cell type examined (Fig. 8). We hypothesize that the RBM45 nucleolar localization in motor neurons limits the protein’s ability to associate with NSBs, and hence, generate nuclear inclusions. Alternatively, spinal cord motor neurons may not form NSBs upon cellular stress. We observed that cytoplasmic sequestration of RBM45 in inclusions is associated with reduced nucleolar RBM45 immunoreactivity (Fig. 8). In contrast to motor neurons, glial cells in the lumbar spinal cord of ALS subjects frequently had RBM45 nuclear inclusions (Fig. 8). Notably, the proportion of glial cells with at least one RBM45 nuclear inclusion was significantly greater than the proportion of glial cells with cytoplasmic RBM45 inclusions (Fig. 8 and Table 2). Similar to our in vitro results and those from the dentate gyrus, we also found that glial SAFB immunoreactivity was reduced in the presence of RBM45 nuclear inclusions. Glial inclusion pathology occurs in various forms of ALS and glial inclusions may contain TDP-43, FUS, SOD1, C9ORF72-associated dipeptide repeat proteins (DPRs), or other ALS-associated proteins [69, 70]. The occurrence of RBM45 inclusions in both the cytoplasm and nucleus of ALS glial cells, together with the diversity of other proteins found in glial inclusions in ALS, underscores the predominant role of glial dysfunction in the pathobiology of ALS and related diseases.

Overall, our results demonstrate that RBM45 associates with NSBs during cellular stress and that chronic stress leads to persistent self-association of RBM45 and nuclear RBM45 inclusion formation. Nuclear RBM45 inclusions are extensively found in the hippocampal dentate gyrus in ALS, FTLD, and AD, as well as in glial cells in ALS spinal cord. The presence of nuclear inclusions is associated with depletion of RBM45 from its normal subcellular milieu and altered distribution of other RBPs. Collectively, our results provide new insights into the biological functions of RBM45, the mechanisms by which RBM45 forms inclusions, and implicate NSBs in the pathogenesis of several neurodegenerative diseases.

## Supplementary information

**Additional file 1: Figure S1.** Assessment of RBM45 association with cytoplasmic stress granules (SGs). HEK293 cells were stained for RBM45 and the SG marker protein G3BP. (a) In untreated cells, the distribution of RBM45 is diffuse and nuclear, while the distribution of G3BP is diffuse and cytoplasmic. (b-g) Treatment with the indicated stressors induced the robust formation of G3BP-positive SGs, which were negative for RBM45. RBM45-positive nuclear stress bodies form in response to each stressor. Arsenite = 1 mM arsenite for 1 h, Heat Shock = 42 °C for 1 h, Serum Dep = serum deprivation for 2 h, CdSO_4_ = 30 μM cadmium sulfate for 2 h, MTX = 20 μM mitoxantrone for 6 h.

**Additional file 2: Figure S2.** Nuclear stress body (NSB) protein levels during conditions of cellular stress. Total protein extracts were prepared from untreated cells, heat shocked cells (42 °C for 2 h), and cells treated with the genotoxic stressor mitoxantrone (MTX; 20 μM for 6 h). Each panel shows the results of loading 10 μg of each extract and blotting for the indicated proteins with actin used as a loading control. (a) RBM45; (b) heat shock factor 1 (HSF1); (c) scaffold attachment factor B (SAFB). No statistically significant differences in the levels of RBM45, HSF1, or SAFB were detected between treatment conditions (*p >* 0.05).

**Additional file 3: Figure S3.** Assessment of TDP-43 and FUS association with nuclear stress bodies (NSBs). (a) HEK293 cells were heat-shocked for 2 h at 42 °C to induce formation of NSBs. Cells were stained for endogeneous RBM45 and TDP-43 or FUS as indicated. Following heat shock, numerous RBM45-positive NSBs become visible in the cell nucleus (arrows) and these do not contain TDP-43 or FUS. Some TDP-43-positive stress granules are visible in the cytoplasm following heat shock and these do not contain RBM45. (b) HEK293 cells were transiently transfected to overexpress HA-tagged TDP-43 or FUS and were then stained for the HA tag and the NSB marker HSF1. Overexpression of TDP-43 was sufficient to induce NSB formation, but NSBs were negative for TDP-43. Neither overexpression of FUS or transfection with a control HA vector resulted in NSB formation. For all images, scale bar = 10 μm.

**Additional file 4: Figure S4.** siRNA targeting of RBM45 and SatIII. HEK293 cells were transfected with the indicated siRNA and transcript levels were measured by real-time PCR. Each bar presents the relative transcript abundance, expressed as a proportion of the corresponding transcript level in untreated cells transfected with a scrambled control siRNA. (a) Evaluation of two unique siRNAs, each targeting RBM45. (b) Evaluation of satellite III (SatIII) knockdown efficiency in untreated and heat shocked (42 °C for 2 h) cells.

**Additional file 5: Figure S5.** Nuclear stress body (NSB) formation during RBM45 and SatIII knockdown. HEK293 cells were transfected with siRNAs targeting RBM45, SatIII, or off-target scrambled siRNAs (control). NSB formation was then assessed by immunocytochemistry following treatment with 1 mM sodium arsenite for 1 h. (a) Effect of off-target, scrambled siRNA (control) on NSB formation. Cells transfected with control siRNAs readily form SAFB, RBM45, and HSF1-positive NSBs following treatment with sodium arsenite. (b) Cells transfected with siRNAs targeting RBM45 show reduced levels of RBM45, but readily form SAFB and HSF1-positive NSBs following cellular stress. (c) Knockdown of SatIII leads to a loss of NSB formation during cellular stress as indicated by the loss of SAFB, RBM45, and HSF1-positive NSBs in cells transfected with SatIII targeting siRNAs. For all images, scale bar = 5 μm.

**Additional file 6: Figure S6.** Subcellular localization of full-length, domain deletion, and mutant NLS RBM45 constructs in untreated cells. (a) Schematic showing functional domains and their position in the full-length RBM45 protein. RRM = RNA recognition motif, HOA = homo-oligomerization domain, NLS = nuclear localization sequence. HEK293 cells were transfected with constructs encoding N-terminally HA-tagged wild-type (WT) or domain-modified forms of RBM45 as indicated. (b) In untreated cells, the distribution of WT RBM45 is diffuse and nuclear. (c) Removal of the RBM45 NLS (∆NLS) leads to cytoplasmic sequestration of RBM45. (d-g) Removal of RRM1 (∆RRM1; d), RRM2 (∆RRM2; e), RRM3 (∆RRM3; f) and the HOA domain (∆HOA; g) do not alter the diffuse, nuclear localization of RBM45. No HSF1-positive NSBs are observed in untreated cells expressing any of the indicated RBM45 constructs. For all images, scale bar = 5 μm.

**Additional file 7: Figure S7.** Quantitative immunocytochemical analysis of RBM45 and SAFB during acute and chronic stress conditions. Nuclear RBM45 and SAFB were quantified by immunocytochemistry and image analysis in untreated and acute and chronically stressed HEK293 cells. To compare RBM45 and SAFB on the same scale, each protein’s signal was converted to Z scores. (a) Nuclear RBM45 levels in untreated and stressed cells. The genotoxic stressor mitoxantrone (MTX, acute = 6 h, 5 μM; chronic = 24 h, 1 μM), the heavy metal stressor cadmium sulfate (CdSO_4_, acute = 2 h, 30 μM; chronic = 24 h, 5 μM), and the oxidative stressor sodium arsenite (Ars, acute = 1 h, 1 mM; chronic = 24 h, 0.1 mM) all significantly reduced the nuclear RBM45 signal compared to untreated cells. (b) Acute MTX treatment, but not chronic MTX treatment significantly reduced the nuclear SAFB signal compared to untreated cells. Acute and chronic CdSO4 treatment significantly reduced the nuclear SAFB signal compared to untreated cells. Acute sodium arsenite treatment, but not chronic sodium arsentie treatment significantly reduced the nuclear SAFB signal compared to untreated cells. For (a) and (b), * = *p* < 1 × 10^− 5^, # = *p* < 0.01.

**Additional file 8: Figure S8.** Characterization of endogeneous RBM45 CRISPR-cas9 edited HEK293 cells. CRISPR-cas9 genome editing was used to generate HEK293 cells expressing N-terminally 2x FLAG-tagged RBM45. (a) RBM45 transcript levels were evaluated by real-time PCR. The barplot presents the mean RBM45 transcript levels relative to unedited cells expressing wild-type RBM45. (b) Immunofluorescence was performed using an antibody to RBM45 in CRISPR-edited HEK293 cells. The results show that the abundance and subcellular distribution of RBM45 protein and HEK293 cell nuclear morphology are not altered by the 2X FLAG tag.
