# Supplementary figures and images for: RBM45 associates with nuclear stress bodies and forms nuclear inclusions during chronic cellular stress and in neurodegenerative diseases
Source: Acta Neuropathol Commun. 2020 Jun 26;8:91. doi: 10.1186/s40478-020-00965-y (PMC7318465; doi:10.1186/s40478-020-00965-y)

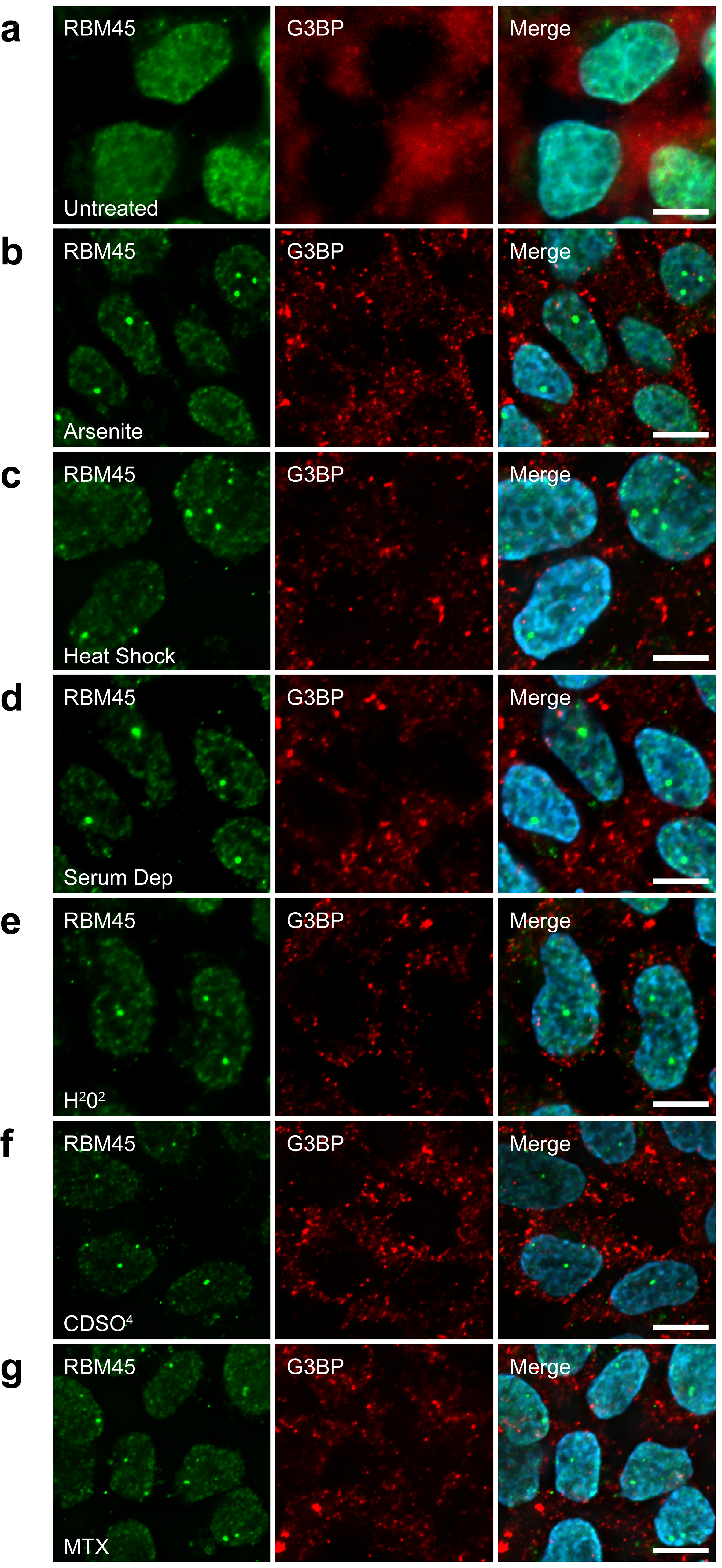

Supplement: Supplementary file 1 — Additional file 1: Figure S1. Assessment of RBM45 association with cytoplasmic stress granules (SGs). HEK293 cells were stained for RBM45 and the SG marker protein G3BP. (a) In untreated cells, the distribution of RBM45 is diffuse and nuclear, while the distribution of G3BP is diffuse and cytoplasmic. (b-g) Treatment with the indicated stressors induced the robust formation of G3BP-positive SGs, which were negative for RBM45. RBM45-positive nuclear stress bodies form in response to each stressor. Arsenite = 1 mM arsenite for 1 h, Heat Shock = 42 °C for 1 h, Serum Dep = serum deprivation for 2 h, CdSO4 = 30 μM cadmium sulfate for 2 h, MTX = 20 μM mitoxantrone for 6 h. [file 40478_2020_965_MOESM1_ESM.tif]

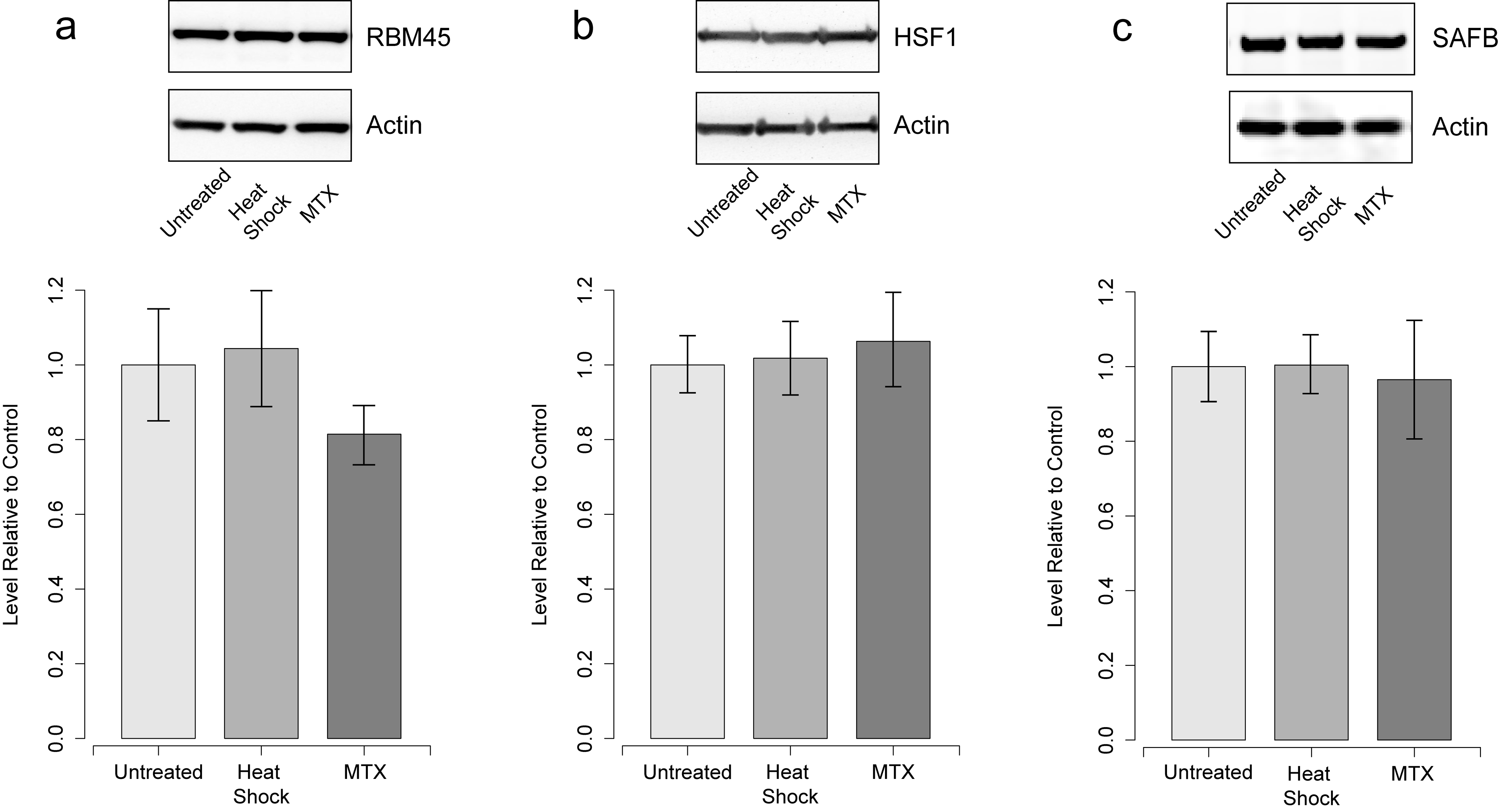

Supplement: Supplementary file 2 — Additional file 2: Figure S2. Nuclear stress body (NSB) protein levels during conditions of cellular stress. Total protein extracts were prepared from untreated cells, heat shocked cells (42 °C for 2 h), and cells treated with the genotoxic stressor mitoxantrone (MTX; 20 μM for 6 h). Each panel shows the results of loading 10 μg of each extract and blotting for the indicated proteins with actin used as a loading control. (a) RBM45; (b) heat shock factor 1 (HSF1); (c) scaffold attachment factor B (SAFB). No statistically significant differences in the levels of RBM45, HSF1, or SAFB were detected between treatment conditions (p > 0.05). [file 40478_2020_965_MOESM2_ESM.tif]

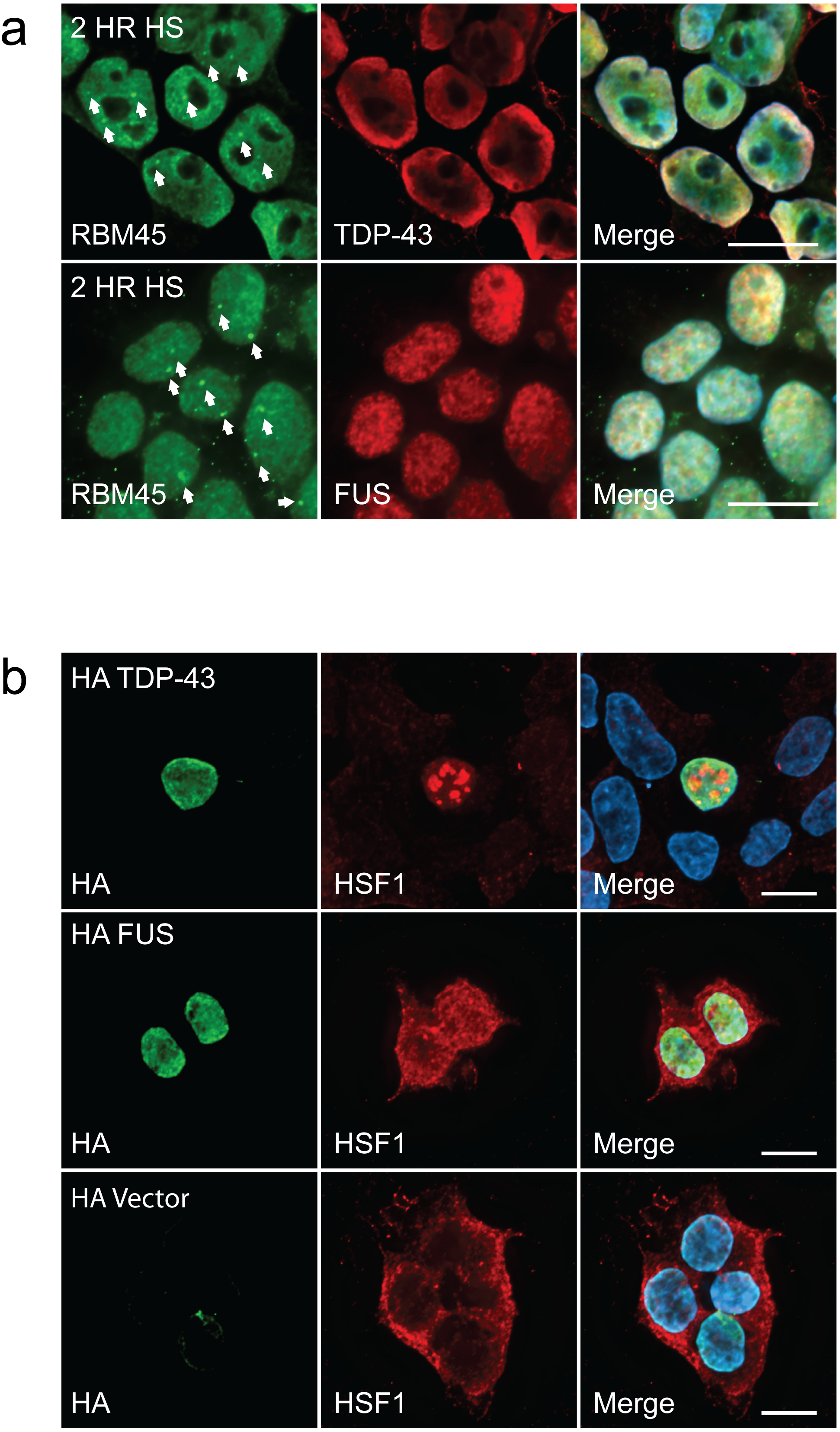

Supplement: Supplementary file 3 — Additional file 3: Figure S3. Assessment of TDP-43 and FUS association with nuclear stress bodies (NSBs). (a) HEK293 cells were heat-shocked for 2 h at 42 °C to induce formation of NSBs. Cells were stained for endogeneous RBM45 and TDP-43 or FUS as indicated. Following heat shock, numerous RBM45-positive NSBs become visible in the cell nucleus (arrows) and these do not contain TDP-43 or FUS. Some TDP-43-positive stress granules are visible in the cytoplasm following heat shock and these do not contain RBM45. (b) HEK293 cells were transiently transfected to overexpress HA-tagged TDP-43 or FUS and were then stained for the HA tag and the NSB marker HSF1. Overexpression of TDP-43 was sufficient to induce NSB formation, but NSBs were negative for TDP-43. Neither overexpression of FUS or transfection with a control HA vector resulted in NSB formation. For all images, scale bar = 10 μm. [file 40478_2020_965_MOESM3_ESM.tif]

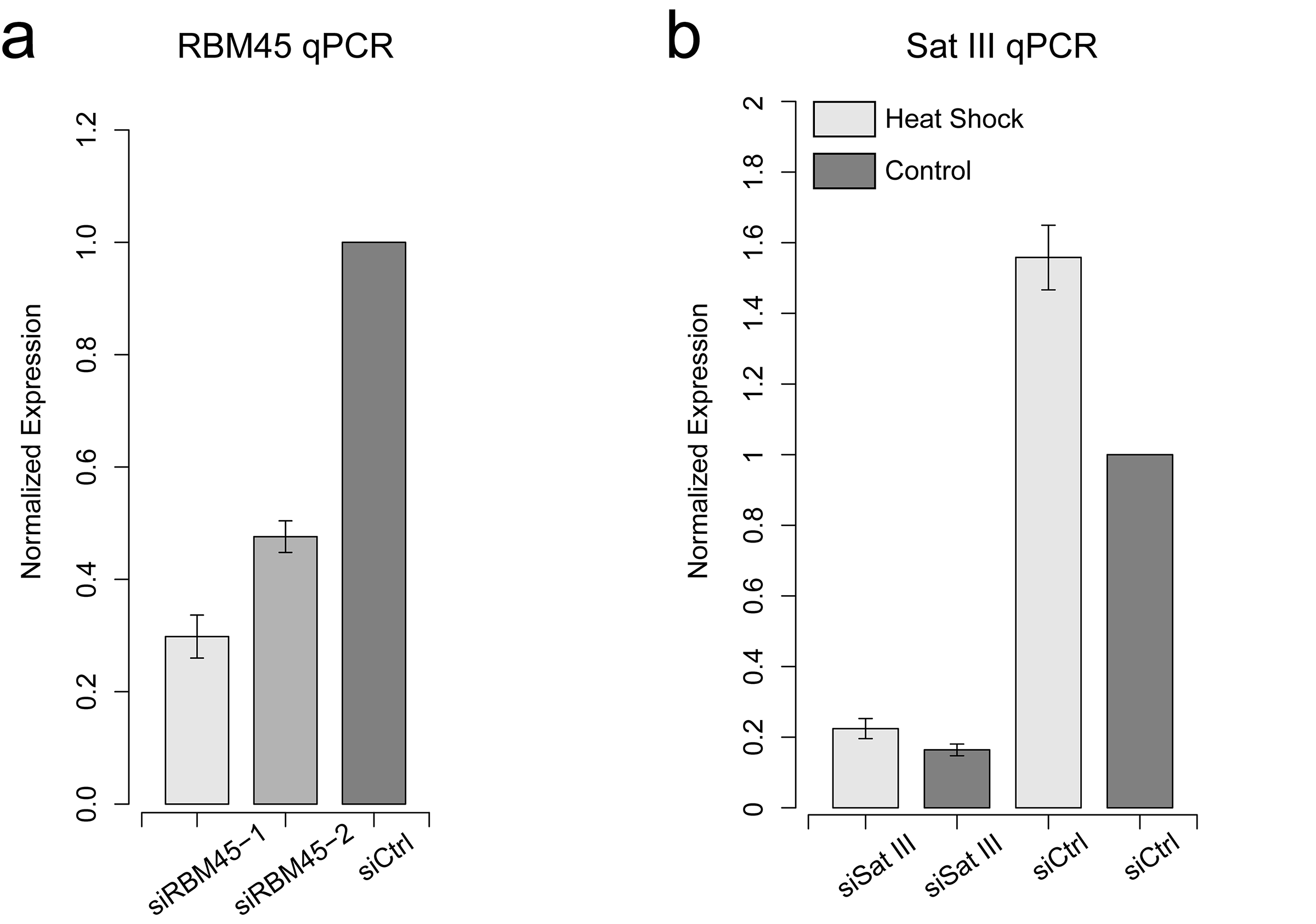

Supplement: Supplementary file 4 — Additional file 4: Figure S4. siRNA targeting of RBM45 and SatIII. HEK293 cells were transfected with the indicated siRNA and transcript levels were measured by real-time PCR. Each bar presents the relative transcript abundance, expressed as a proportion of the corresponding transcript level in untreated cells transfected with a scrambled control siRNA. (a) Evaluation of two unique siRNAs, each targeting RBM45. (b) Evaluation of satellite III (SatIII) knockdown efficiency in untreated and heat shocked (42 °C for 2 h) cells. [file 40478_2020_965_MOESM4_ESM.tif]

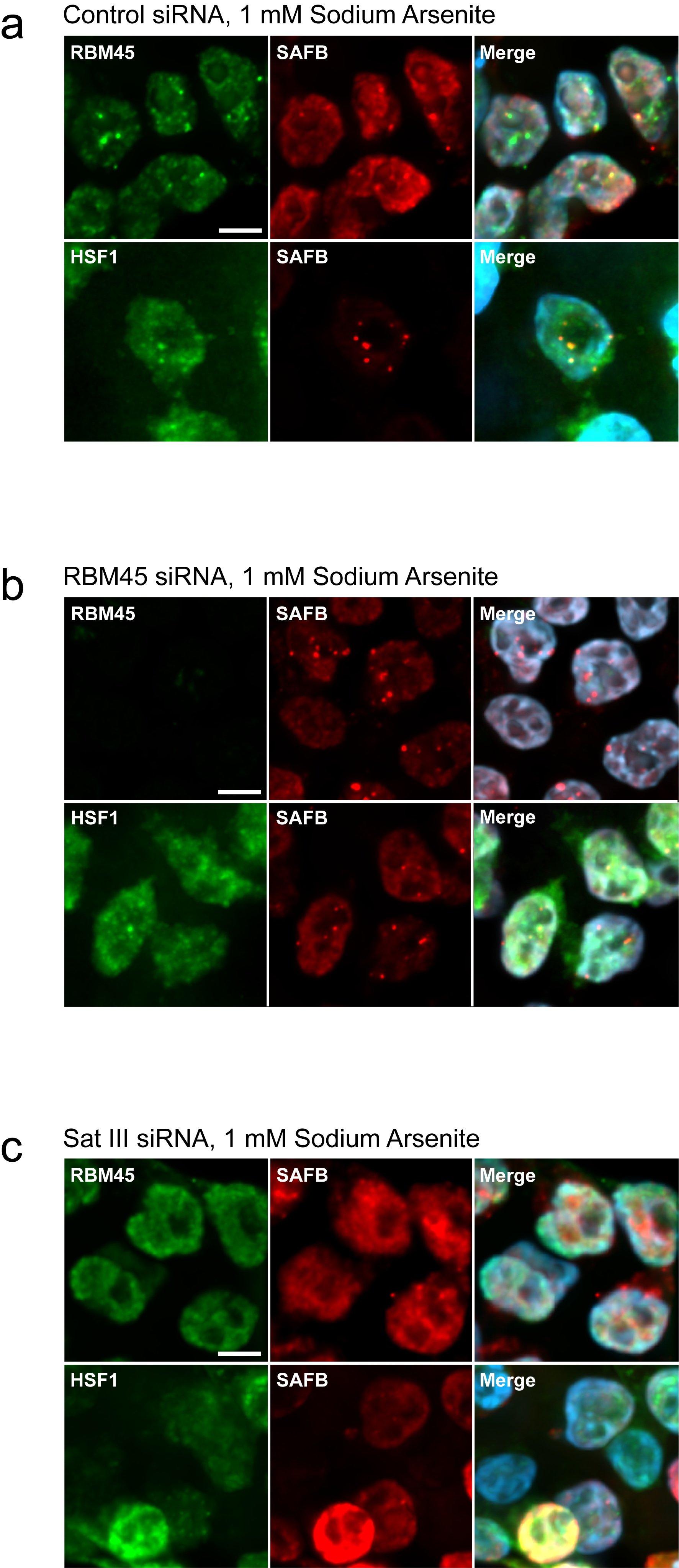

Supplement: Supplementary file 5 — Additional file 5: Figure S5. Nuclear stress body (NSB) formation during RBM45 and SatIII knockdown. HEK293 cells were transfected with siRNAs targeting RBM45, SatIII, or off-target scrambled siRNAs (control). NSB formation was then assessed by immunocytochemistry following treatment with 1 mM sodium arsenite for 1 h. (a) Effect of off-target, scrambled siRNA (control) on NSB formation. Cells transfected with control siRNAs readily form SAFB, RBM45, and HSF1-positive NSBs following treatment with sodium arsenite. (b) Cells transfected with siRNAs targeting RBM45 show reduced levels of RBM45, but readily form SAFB and HSF1-positive NSBs following cellular stress. (c) Knockdown of SatIII leads to a loss of NSB formation during cellular stress as indicated by the loss of SAFB, RBM45, and HSF1-positive NSBs in cells transfected with SatIII targeting siRNAs. For all images, scale bar = 5 μm. [file 40478_2020_965_MOESM5_ESM.tif]

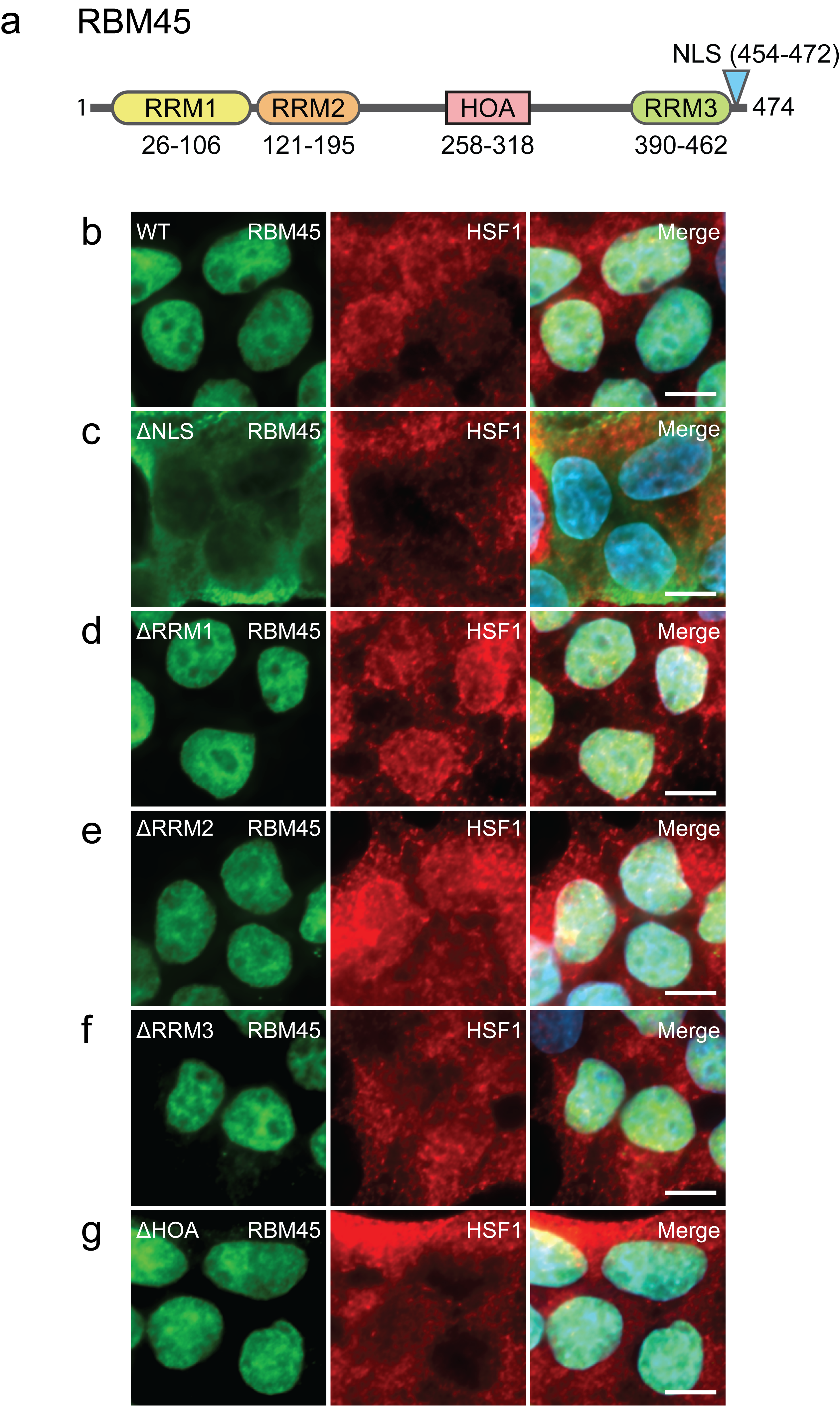

Supplement: Supplementary file 6 — Additional file 6: Figure S6. Subcellular localization of full-length, domain deletion, and mutant NLS RBM45 constructs in untreated cells. (a) Schematic showing functional domains and their position in the full-length RBM45 protein. RRM = RNA recognition motif, HOA = homo-oligomerization domain, NLS = nuclear localization sequence. HEK293 cells were transfected with constructs encoding N-terminally HA-tagged wild-type (WT) or domain-modified forms of RBM45 as indicated. (b) In untreated cells, the distribution of WT RBM45 is diffuse and nuclear. (c) Removal of the RBM45 NLS (∆NLS) leads to cytoplasmic sequestration of RBM45. (d-g) Removal of RRM1 (∆RRM1; d), RRM2 (∆RRM2; e), RRM3 (∆RRM3; f) and the HOA domain (∆HOA; g) do not alter the diffuse, nuclear localization of RBM45. No HSF1-positive NSBs are observed in untreated cells expressing any of the indicated RBM45 constructs. For all images, scale bar = 5 μm. [file 40478_2020_965_MOESM6_ESM.tif]

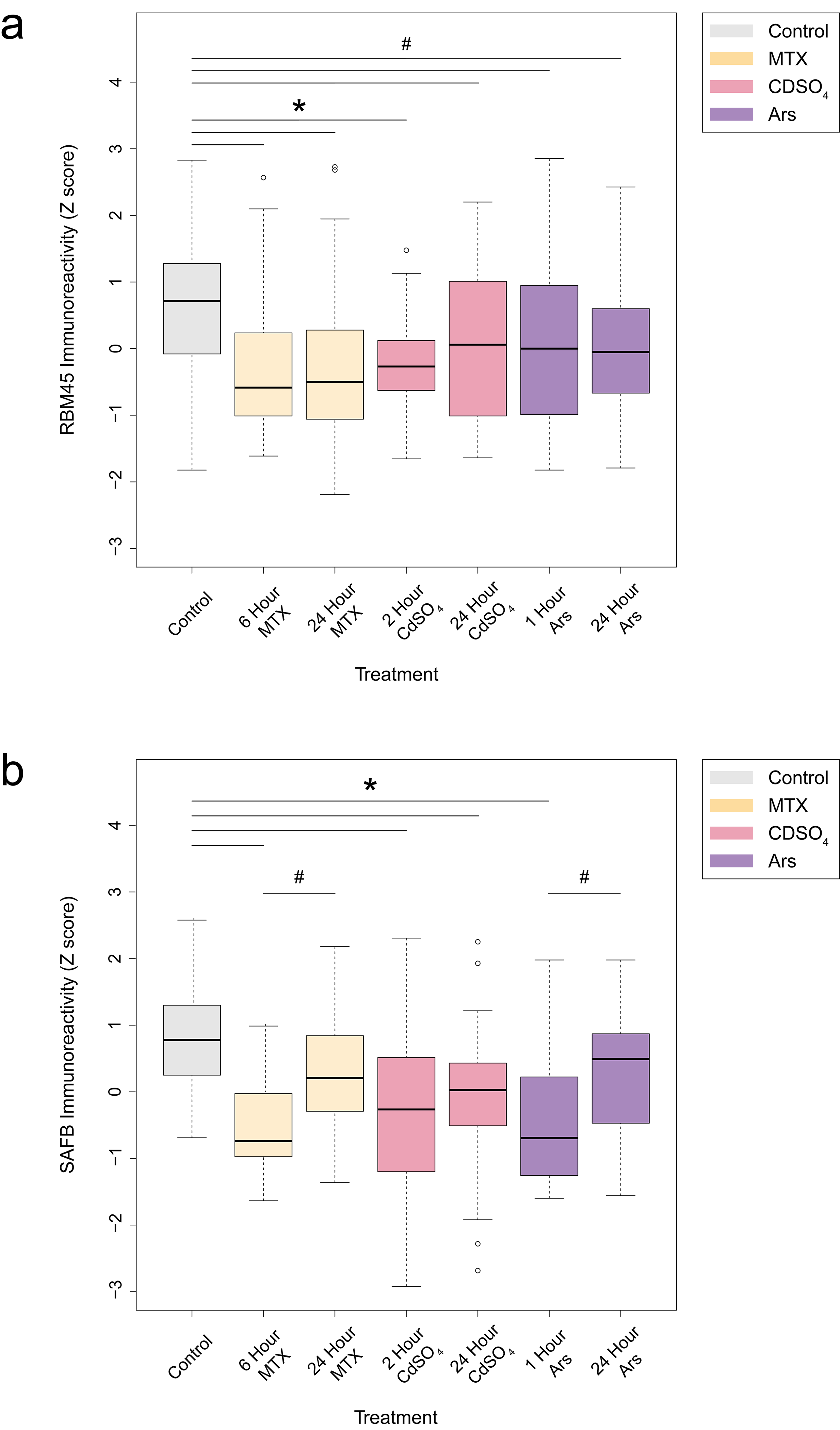

Supplement: Supplementary file 7 — Additional file 7: Figure S7. Quantitative immunocytochemical analysis of RBM45 and SAFB during acute and chronic stress conditions. Nuclear RBM45 and SAFB were quantified by immunocytochemistry and image analysis in untreated and acute and chronically stressed HEK293 cells. To compare RBM45 and SAFB on the same scale, each protein’s signal was converted to Z scores. (a) Nuclear RBM45 levels in untreated and stressed cells. The genotoxic stressor mitoxantrone (MTX, acute = 6 h, 5 μM; chronic = 24 h, 1 μM), the heavy metal stressor cadmium sulfate (CdSO4, acute = 2 h, 30 μM; chronic = 24 h, 5 μM), and the oxidative stressor sodium arsenite (Ars, acute = 1 h, 1 mM; chronic = 24 h, 0.1 mM) all significantly reduced the nuclear RBM45 signal compared to untreated cells. (b) Acute MTX treatment, but not chronic MTX treatment significantly reduced the nuclear SAFB signal compared to untreated cells. Acute and chronic CdSO4 treatment significantly reduced the nuclear SAFB signal compared to untreated cells. Acute sodium arsenite treatment, but not chronic sodium arsentie treatment significantly reduced the nuclear SAFB signal compared to untreated cells. For (a) and (b), * = p < 1 × 10− 5, # = p < 0.01. [file 40478_2020_965_MOESM7_ESM.tif]

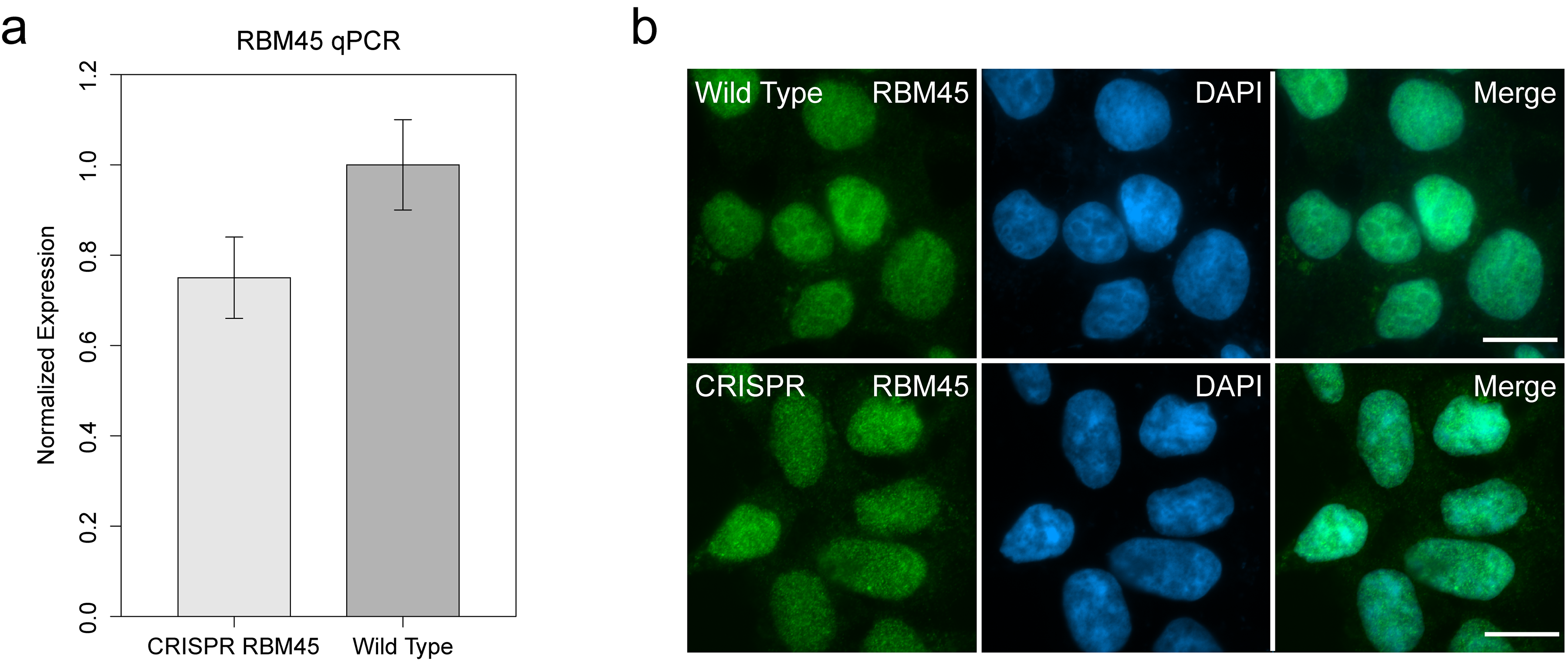

Supplement: Supplementary file 8 — Additional file 8: Figure S8. Characterization of endogeneous RBM45 CRISPR-cas9 edited HEK293 cells. CRISPR-cas9 genome editing was used to generate HEK293 cells expressing N-terminally 2x FLAG-tagged RBM45. (a) RBM45 transcript levels were evaluated by real-time PCR. The barplot presents the mean RBM45 transcript levels relative to unedited cells expressing wild-type RBM45. (b) Immunofluorescence was performed using an antibody to RBM45 in CRISPR-edited HEK293 cells. The results show that the abundance and subcellular distribution of RBM45 protein and HEK293 cell nuclear morphology are not altered by the 2X FLAG tag. [file 40478_2020_965_MOESM8_ESM.tif]
